# Structure-Guided Design of d-Galactal Derivatives with High Affinity and Selectivity for the Galectin-8 N-Terminal Domain

**DOI:** 10.1021/acsmedchemlett.1c00371

**Published:** 2021-11-02

**Authors:** Mujtaba Hassan, Floriane Baussière, Samo Guzelj, Anders P. Sundin, Maria Håkansson, Rebeka Kovačič, Hakon Leffler, Tihomir Tomašič, Marko Anderluh, Žiga Jakopin, Ulf J. Nilsson

**Affiliations:** † Centre for Analysis and Synthesis, Department of Chemistry, Lund University, Box 124, SE-221 00 Lund, Sweden; ‡ Department of Medicinal Chemistry, Faculty of Pharmacy, University of Ljubljana, Aškerčeva 7, 1000 Ljubljana, Slovenia; § SARomics Biostructures AB, Medicon Village, SE-223 63 Lund, Sweden; ∥ Department of Laboratory Medicine, Section MIG, Lund University BMC-C1228b, Klinikgatan 28, 221 84 Lund, Sweden

**Keywords:** Galectin-8N, d-galactal, benzimidazole, selectivity, X-ray crystallography, cytokine secretion

## Abstract

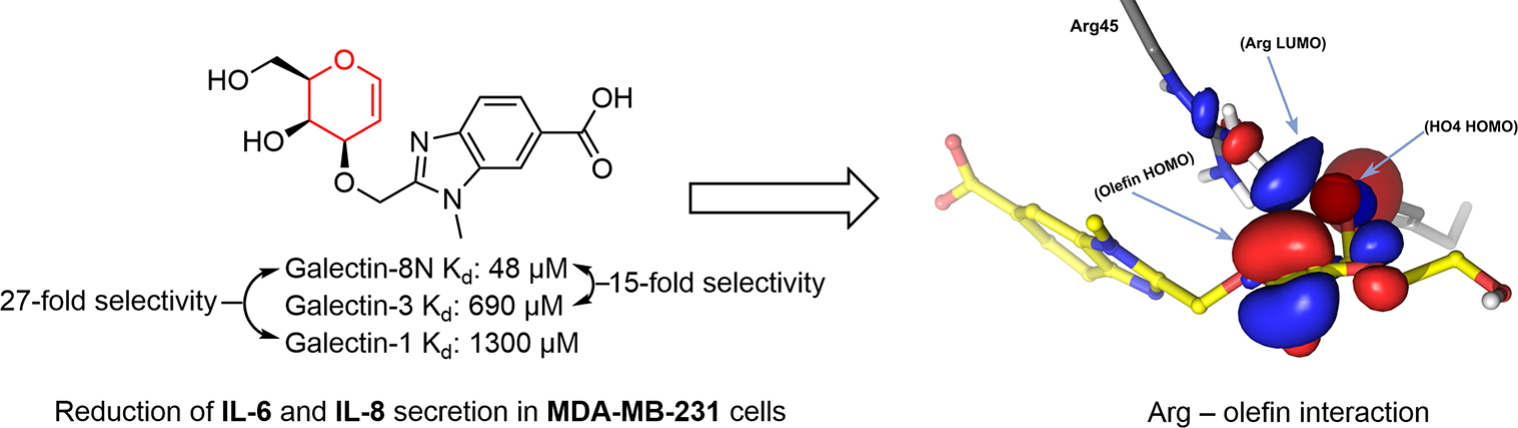

Galectin-8 is a carbohydrate-binding protein that plays a crucial role in tumor progression and metastasis, antibacterial autophagy, modulation of the immune system, and bone remodeling. The design, synthesis, and protein affinity evaluation of a set of C-3 substituted benzimidazole and quinoline d-galactal derivatives identified a d-galactal-benzimidazole hybrid as a selective ligand for the galectin-8 N-terminal domain (galectin-8N), with a *K*_d_ of 48 μM and 15-fold selectivity over galectin-3 and even better selectivity over the other mammalian galectins. X-ray structural analysis of galectin-8N in complex with one benzimidazole- and one quinoline-galactal derivative at 1.52 and 2.1 Å together with molecular dynamics simulations and quantum mechanical calculations of galectin-8N in complex with the benzimidazole derivative revealed orbital overlap between a NH LUMO of Arg45 with electron rich HOMOs of the olefin and O4 of the d-galactal. Such overlap is hypothesized to contribute to the high affinity of the d-galactal-derived ligands for galectin-8N. A (3-(4,5-dimethylthiazol-2-yl)-5-(3- carboxymethoxyphenyl)-2-(4-sulfophenyl)-2*H*-tetrazolium) (MTS) assay evaluation of the d-galactal-benzimidazole hybrid and an analogous galactoside derivative on a panel of cell lines with MTS assay showed no effect on cell viability up to 100 μM concentration. A subsequent functional assay using the MDA-MB-231 cell line demonstrated that the d-galactal-benzimidazole hybrid and the analogous galactoside derivative reduced the secretion of the proinflammatory cytokines interleukin-6 (IL-6) and IL-8 in a dose-dependent manner. Therefore, these compounds represent potential probes for galectin-8N pharmacology investigations and possibly promising leads for the design and synthesis of potent and selective galectin-8 inhibitors as potential antitumor and anti-inflammatory agents.

Galectins are a family of proteins that bind β-d-galactopyranoside-containing glycoconjugates through their canonical carbohydrate recognition domains (CRDs).^1^ They are synthesized in the cytosol, and spend most of their lifetime in the cytosol or the nucleus where they interact with a myriad of cytosolic and nuclear targets.^2^ Galectins also reach the extracellular matrix via a nonclassical secretion where they cross-link the cell-surface glycoproteins and glycolipids and form lattices.^3^ In doing so, galectins influence several properties of the cell-surface glycoconjugates such as their membrane residence time, cellular trafficking, and sorting. Through interacting with intracellular and extracellular targets, galectins take part in a myriad of cellular functions including organizing the cell surface, guiding endocytic pathways, regulating cell adhesion, and targeting disrupted vesicles in the cytosol.^4,5^ Galectins exist as dimers, or monomers that oligomerize upon binding, or divalent proteins with two CRDs at the N- and C-terminals.^6^ Galectin-8 is a divalent galectin found in the liver, the kidney, the cardiac muscle, the lung, and the brain.^7^ Due to its glycan binding capacity, it is involved in the regulation of cell growth, cell adhesion, cell migration, embryogenesis, apoptosis, and modulation of the immune responses.^7^ Importantly, galectin-8 is involved in vascular endothelial growth factor C (VEGF-C) mediated pathological lymphangiogenesis, which is associated with several pathological conditions such as tumor growth and metastasis, solid organ graft rejection, and corneal inflammation.^8^ Notably, galectin-8 is upregulated in several cancer tissues including breast, prostate, bladder, kidney, and lung tissues.^7^ In addition, galectin-8 also plays important roles in autoimmune and inflammatory disorders, antibacterial autophagy, osteoporosis, and bone loss.^9−11^ Its involvement in several pathological conditions attests to the importance of selective galectin-8 inhibitors as research tools and potential antitumor and anti-inflammatory drugs.

The two CRDs of galectin-8 share the same amino acids forming the galactose-binding site, namely, Trp86, His65, Asn67, Arg69, Asn79, and Glu89. However, there are major differences between the two CRDs, such as the presence of Arg45, Arg59, Tyr141, and Gln47 in the N-terminal CRD (galectin-8N) and the presence of Ser255, Asn257, and Asn348 in the C-terminal CRD (galectin-8C).^12^ The Arg59 side chain is responsible for the preferential binding of galectin-8N to anionic glycans, such as 3′-*O*-sulfate/3′-*O*-sialylated lactosides, via salt bridges.^13,14^ Moreover, galectin-8N binds a broader spectrum of glycans with higher affinity compared to the C-terminal (galectin-8C).^15^ As the inhibition of one CRD is hypothesized to be sufficient to block the biological activity of galectin-8,^16^ we directed our efforts toward the design and synthesis of galectin-8N ligands.

Achieving selectivity for galectin-8N over the other mammalian galectins has always been a challenging task, due to the substantial similarity of different galectin CRDs and even between 8N and 8C CRDs. The previously reported synthetic galectin-8N ligands include a coumarin-galactoside derivative (*K*_d_ = 200 μM),^17^ a methyl β-d-galactomalonyl phenyl ester (*K*_d_ = 5.7 μM),^18^ a quinoline-galactoside (*K*_d_ = 1.5 μM),^19^ and a recently reported benzimidazole-galactoside **1** (*K*_d_ = 1.8 μM)^20^ (Figure 1). Of particular note is a recently reported tricyclic galactose-benzene hybrid **2** (*K*_d_ = 180 μM) which has about 2-fold selectivity over galectin-1 and a high 23-fold selectivity over galectin-3 (Figure 1).^21^ Published docking analyses of compound **2** in complex with galectin-8N suggested that the endocyclic double bond at the junction between the 3,4-dihydropyran ring and the adjacent phenyl ring of **2** lies within 3.5 Å to the electropositive side chain guanidinium ion of Arg45.^21^ This distance would allow for an interaction between the double bond and Arg45. Furthermore, the phenyl moieties of compound **2** were solvent-exposed and not in contact with galectin-8N amino acid side chains.^21^ Therefore, we hypothesized that the endocyclic double bond of compound **2** possibly accounts for the higher affinity and selectivity of the compound for galectin-8N through interaction with Arg45 while none of its phenyl rings engage in specific interactions with the neighboring residues. Hence, the d-galactal **3** represents the minimum active fragment of compound **2**.

**Figure 1:**
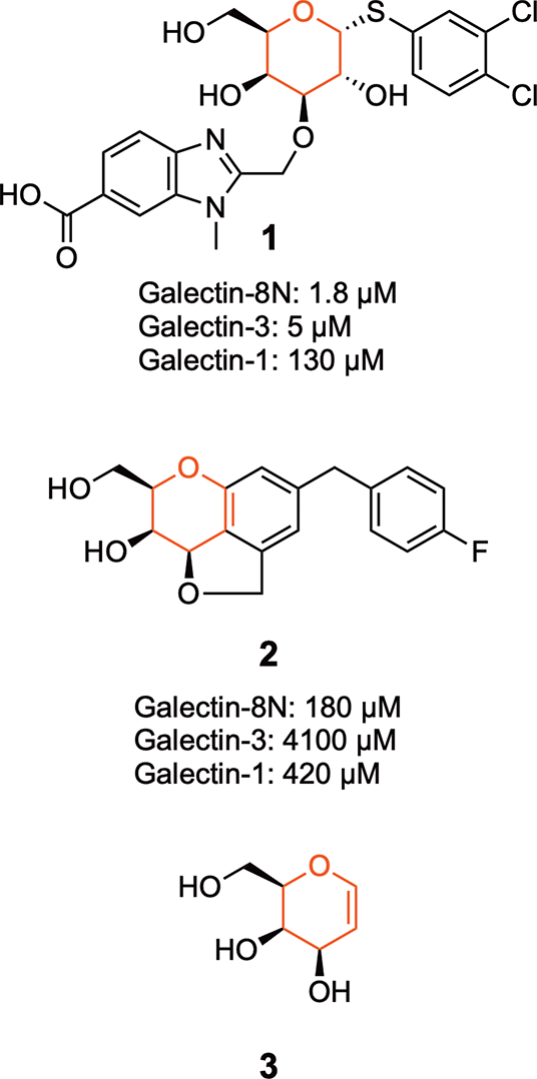
Structures and *K*_d_ values of the benzimidazole-galactoside **1**, the tricyclic carbohydrate-benzene hybrid **2**, and d-galactal **3**.

Moreover, the chemistry of the 1,2-glycals is well-established with a plethora of reactions that can be conducted at the different carbons of d-galactal.^22−27^ Therefore, d-galactal (**3**) represents a promising galactose mimic for the iterative design and optimization of galectin-8N ligands. Herein, we report the design, synthesis, and biological evaluation of C-3 substituted d-galactal derivatives as galectin-8N ligands with high selectivity over the other mammalian galectins.

## Results and Discussion

### Inhibitor Design and Synthesis

**Scheme 1:**
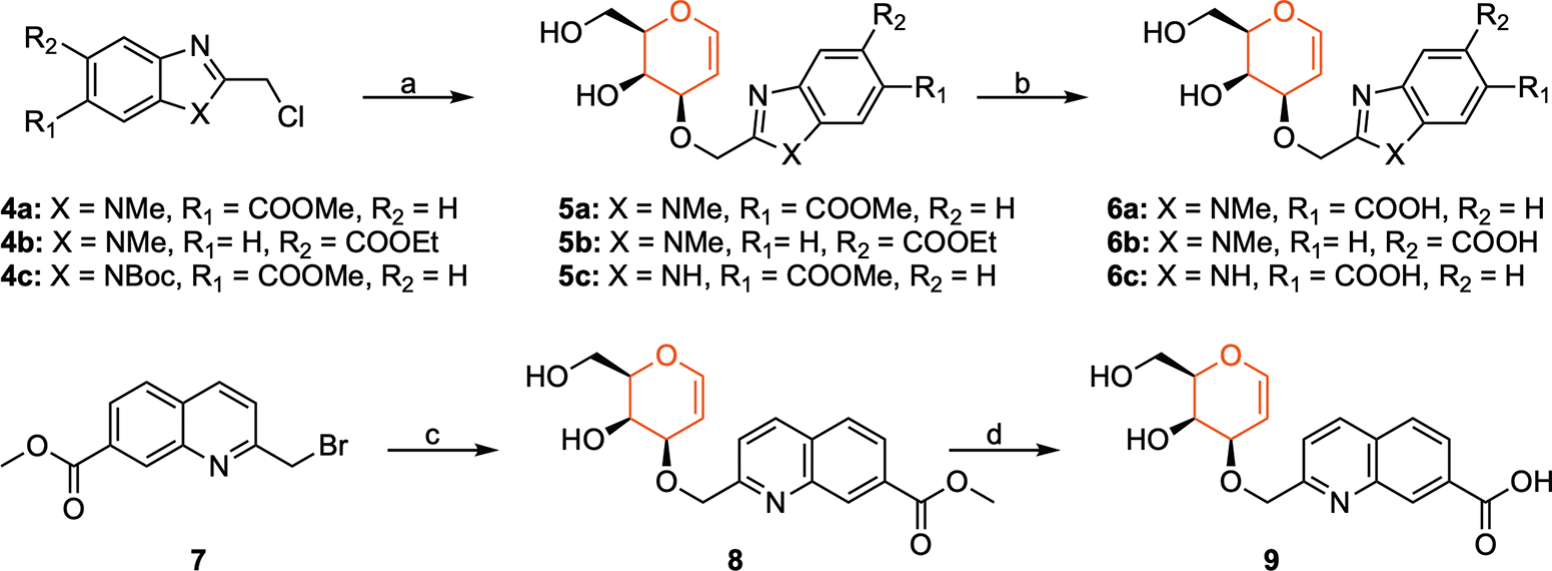
(a) d-Galactal (**3**), Bu_2_SnO, *n*-Bu_4_NI, PhMe, MeCN, MW, 120 °C, 30 min (44–67%). (b) KOH, EtOH, H_2_O, 80 °C, 6 h (41–76%). (c) Bu_2_SnO, *n*-Bu_4_Br, DIPEA, 80 °C, 90 min, 50%. (d) KOH, EtOH, H_2_O, 80 °C, 6 h, 83%.

Based on the reported binding affinities of the benzimidazole and quinoline-galactosides to galectin-8N, we hypothesized that their d-galactal featuring counterpart might result in ligands that bind galectin-8N with improved affinity. Hence, d-galactal was alkylated with benzimidazolyl- and quinolyl-methyl moieties at O3. The benzimidazolylmethyl chlorides **4a**–**4c** and the quinolylmethyl chloride **7** were synthesized as previously reported.^19^ Alkylation of the d-galactal **3** with the benzimidazolylmethyl chlorides **4a**–**4c** and the quinolylmethyl chloride **7** via stannylene-mediated 3-*O*-alkylation of the d-galactal **3** afforded the benzimidazole methyl esters **5a**–**5c** and the quinoline methyl ester **8** (Scheme 1).^23,28^ The subsequent alkaline hydrolysis of the esters produced the benzimidazole carboxylates **6a**–**6c** and the quinoline carboxylate **9** (Scheme 1).

### Galectin Affinity Evaluation

The binding affinities of the benzimidazoles **5a**–**5c** and **6a**–**6c** and the quinolines **8** and **9** for galectins-1, -3, -4, -7, -8C, -8N, -9C, and -9N were determined in a competitive fluorescence polarization assay as previously described.^29−31^ The d-galactal **3** had a 5-fold higher affinity for galectin-8N compared to the methyl β-d-galactopyranoside (Table 1), which supports our hypothesis that the endocyclic double bond of d-galactal **3** plays a role in increasing the binding affinity to galectin-8N. All synthesized d-galactal derivatives had higher binding affinities for galectin-8N than d-galactal **3**. Except for compounds **5b** and **6b**, the carboxylic acid derivatives had a 4–8-fold higher affinity for galectin-8N compared to their ester counterparts. In addition, all d-galactal derivatives showed a 2–7-fold higher affinity for galectin-8N compared to the previously published benzimidazole and quinoline methyl β-d-galactosides.^19^ Compounds **6a**, **6c**, and **9** had nearly identical *K*_d_ values for galectin-8N, with compound **6c** exhibiting the highest gain in binding affinity compared to its galactoside equivalent with a *K*_d_ of 46 μM. In terms of selectivity, except for compounds **5b** and **6b,** the d-galactal derivatives were more than 2-fold more selective for galectin-8N over other mammalian galectins, with compound **6a** being the most selective galectin-8N ligand to date with 15-fold selectivity over galectin-3, 27-fold selectivity over galectin-1, and even higher selectivity over the other mammalian galectins (Table 1).

**Table 1:** *K*_d_ Values of Compounds **5a**–**5c**, **6a**–**6c**, **8**, and **9** (μM)^a^

	galectins (N = N-terminal domain, C = C-terminal domain)									
compd	1	2	3	4N	4C	**7**	8N	8C	9N	9C
**2**(21)	420	NA^b^	4100	>1000	>3000	NA^b^	180	NA^b^	>1000	>1000
**3**	1600 ± 140	NA^b^	3300 ± 650	2400 ± 13	5300	NA^b^	1300 ± 72	4100 ± 470	NB^c^	4800
methyl β-d-galactoside^19,31,32^	>10 000	13000	4400	6600	1000	NA^b^	6300	>30 000	3300	8600 ± 7309
**5a**	2200 ± 138	NA^b^	1300 ± 130	1700 ± 60	2600 ± 230	NA^b^	400 ± 34	3000 ± 380	NB^c^	NA^b^
**5b**	840 ± 20	NA^b^	680 ± 37	NA^b^	NA^b^	NA^b^	690 ± 53	1500 ± 180	NA^b^	NA^b^
**5c**	NB^c^	NA^b^	1200 ± 287	3200	2500	NA^b^	180 ± 19	3000 ± 300	NB^c^	NB^c^
**6a**	1300 ± 130	1400 ± 24	690 ± 30	1400 ± 30	1700 ± 150	NB^c^	48 ± 4	4000 ± 500	1400 ± 57	NB^c^
**6b**	1100 ± 87	NA^b^	770 ± 129	3600	2500 ± 300	NA^b^	810 ± 54	3000 ± 30	NB^c^	NB^c^
**6c**	NB^c^	990 ± 170	550 ± 32	1400 ± 230	1900 ± 400	NB^c^	46 ± 4	NA^b^	NB^c^	2500
**8**	NB^c^	NB^c^	1700 ± 40	1300 ± 12	3700 ± 250	NA^b^	230 ± 16	NB^c^	1000 ± 51	NB^c^
**9**	3600	1100 ± 200	590 ± 78	NB^c^	2800 ± 770	NB^c^	48 ± 6	4700 ± 22	1800 ± 160	NB^c^

a Results represent the mean ± SEM of *n* = 4–8.

b Not available.

c Nonbinding up to the highest tested concentration of 1500 μM.

### Structural Analysis

**Figure 2:**
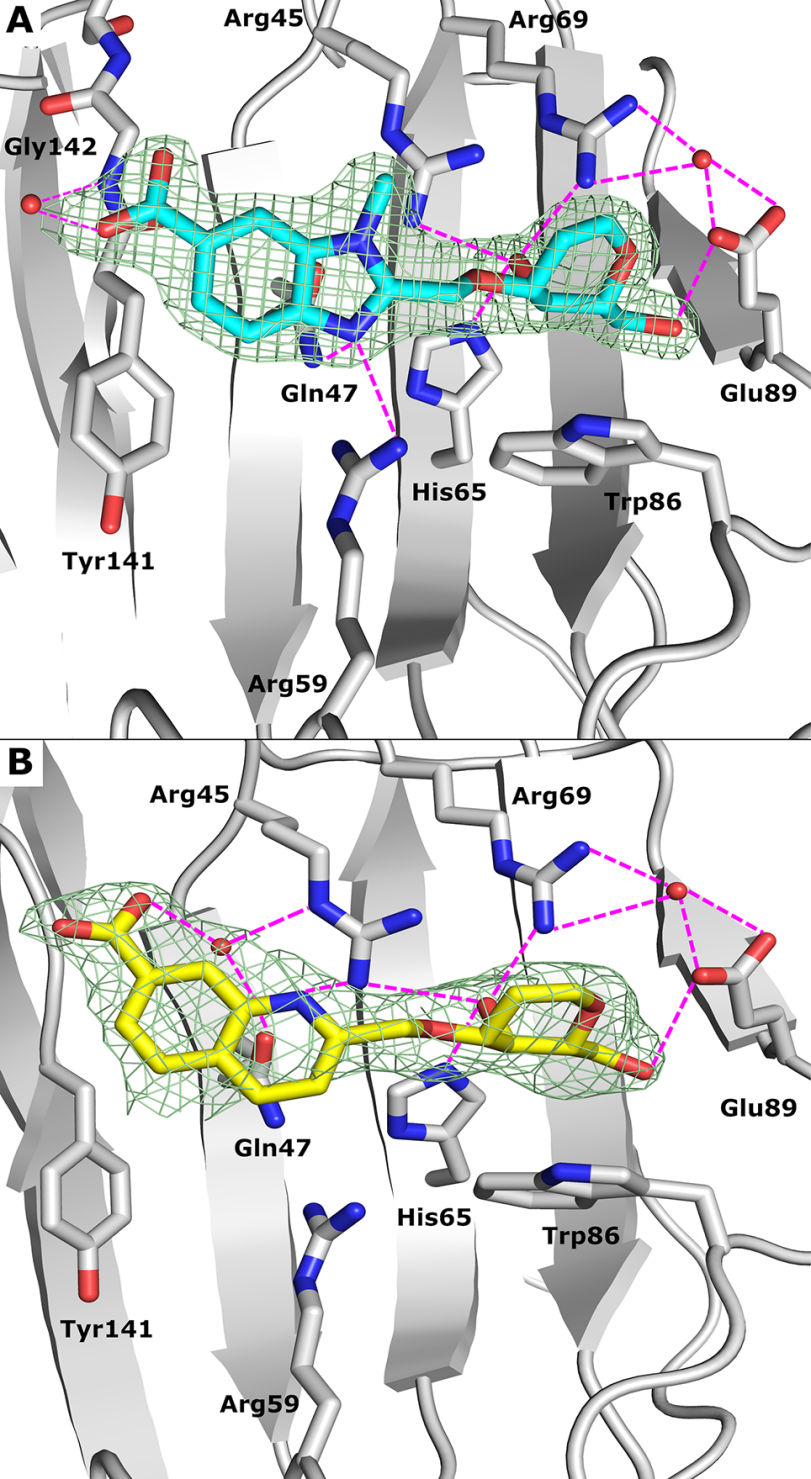
Electron densities 2|F_0_| – |F_c_|α_c_ contoured at 1σ for compounds (A) **6a** (PDB ID: 7P1M; cyan cartoon representation, 1.52 Å) and (B) **9** (PDB ID: 7P11; yellow cartoon representation, 2.1 Å) in complex with galectin-8N. The magenta dashed lines indicate the polar interactions between the ligands and galectin-8N.

To investigate the binding modes of compounds **6a** and **9** for galectin-8N, we solved their X-ray crystal structures in complex with galectin-8N according to the previously published protocol.^20^ Galectin-8N was first cocrystallized with lactose, and then the ligands were soaked into galectin-8N-lactose crystals, followed by collection of X-ray diffraction data at 1.52 and 2.1 Å resolution for the galectin-8N–**6a** complex and galectin-8N–**9** complex, respectively. Both complexes showed that the O4 and O6 of the d-galactal portion bind to galectin-8N in a similar manner as the corresponding oxygens in the d-galactosides. Namely, the O4 of the d-galactal is involved in a hydrogen-bonding network with Arg45, Arg69, and His65. Further, the O6 of the d-galactal engages in hydrogen bonding interactions with Asn79 and Glu89, while the O3 engages in a hydrogen bonding interaction with Arg45 (Figure 2). As for the galectin-8N–**6a** complex, the five-membered ring of the benzimidazole ring is placed in a favorable position to engage in cation−π stacking with Arg45 while the basic nitrogen of the benzimidazole engages in a dipole–dipole interaction with Gln 47 and Arg59. The carboxylate moiety of the benzimidazole establishes a water-mediated hydrogen bond with Gly142. As for the galectin-8N–**9** complex, the quinoline ring is placed in a favorable position to establish an interaction with Arg45 while the carboxylate moiety establishes a water-mediated hydrogen bond with Gln47 and Arg45. The endocyclic double bond of the d-galactal moiety in both complexes is placed in proximity to the guanidinium side chains of Arg45 and Arg69 (Figure 2).

### Molecular Modeling

**Figure 3:**
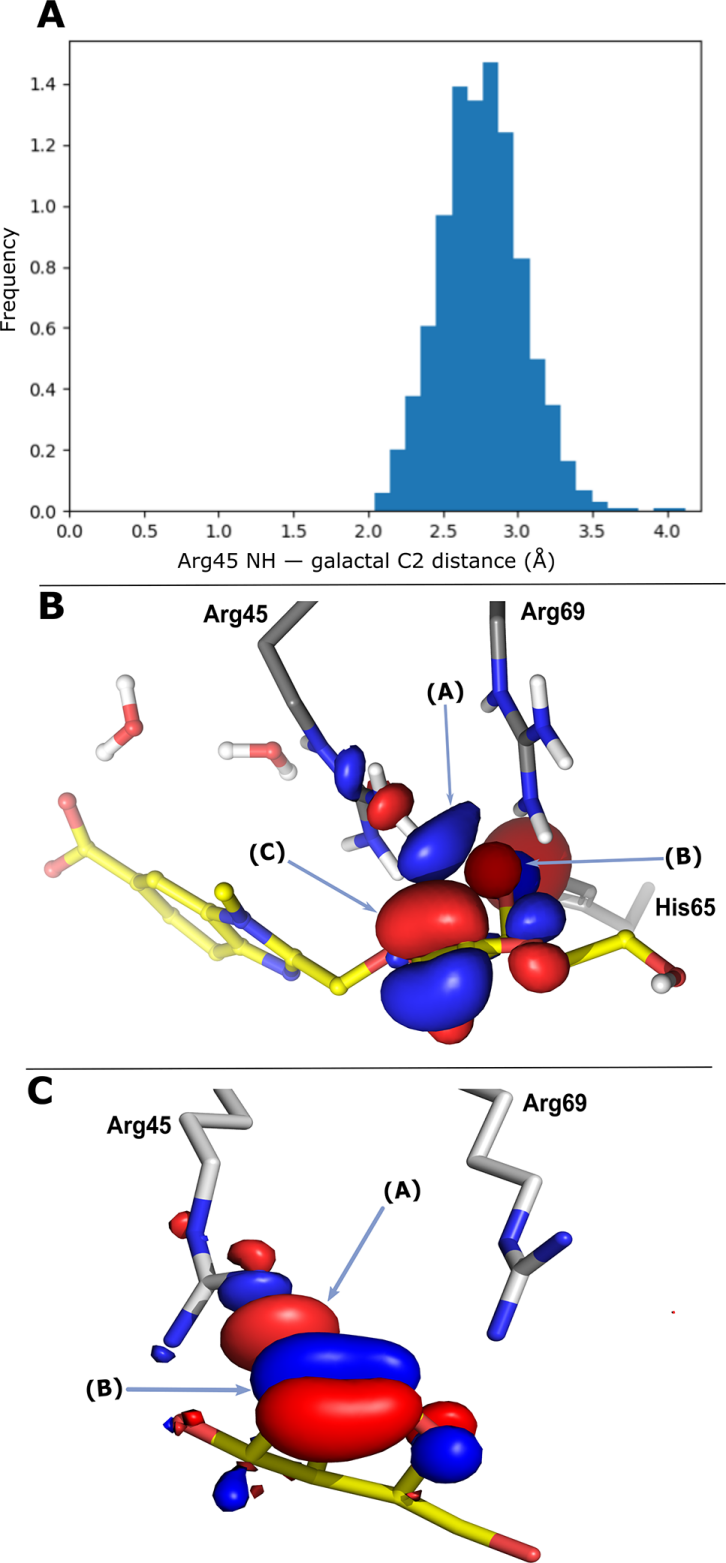
(A) Distance distribution between O4 of the d-galactal and NH of Arg45 during the 300 ns molecular dynamic simulation. (B) Quantum mechanical calculations on a representative MD snapshot (265 ns) of the galectin-8N–**6a** complex using Jaguar (Schrodinger suite). The calculations revealed that the LUMO of Arg45 of galectin-8N (A, depicted in blue) interacts with the HOMO of O4 (B, depicted in red), as well as the HOMO of the olefin of the d-galactal ring (C, depicted in red) of compound **6a**. These interactions are unique and account for the higher affinity of **6a** for galectin-8N. (C) Quantum mechanical calculations on a representative MD snapshot (202 ns) of the galectin-8N–**3** (in yellow sticks) complex using Jaguar (Schrodinger suite). The LUMO of Arg45 of galectin-8N (A, depicted in red) interacts with the HOMO of the d-galactal **3** olefin (B, depicted in blue).

In order to test the hypothesis that the olefin of the d-galactal ring interacts with guanidinium side chains, a 300 ns molecular dynamics simulation of the galectin-8N–**6a** X-ray complex was performed. A representative MD snapshot at 265 ns, with a C2 to Arg45 distance similar to the 2.74 Å in the crystal structure, was selected for QM calculations. The molecular orbitals of **6a** and the neighboring amino acid side chains were calculated using HF/4-21G*. A NH LUMO of Arg45 overlapped with the HOMOs of O4 and the d-galactal double bond (Figure 3, panels A and B). A 300 ns molecular dynamics snapshot (202 ns) of d-galactal **3** in complex with galectin-8N, followed by QM analysis as for **6a**, revealed a similar corresponding orbital overlap. d-Galactal **3** is similar in shape to methyl β-d-galactoside, with three corresponding hydroxyl groups (at C3, C4, and C6), but it binds a factor of 5 better than methyl β-d-galactoside (Figure 3, panel C). Hence, a favorable interaction between one of the Arg45 protons and the d-galactal double bond likely contributes to improved affinity of galectin-8N for d-galactal derivatives.

**Figure 4:**
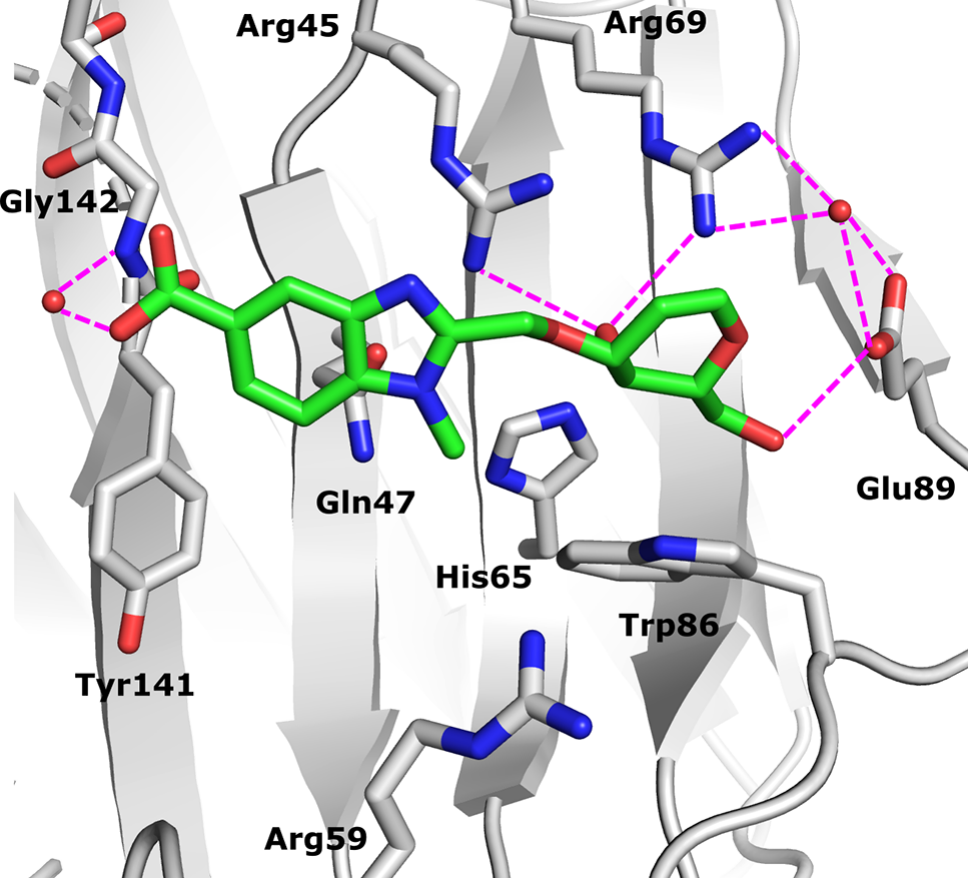
Representative molecular dynamics simulation snapshot of **6b** (in green sticks) in complex with galectin-8N. The benzimidazole N3 of **6b** formed an electrostatic interaction with a guanidinium side chain hydrogen, while carboxylate moiety of **6b** formed a water-mediated hydrogen bond with the backbone N–H of Gly142.

Furthermore, molecular dynamics simulations were also performed with **6b** (200 ns) to understand the affinity difference between compounds **6a** and **6b** for galectin-8N. The *N*-methyl of **6b** in complex with galectin-8N was oriented facing Arg59 regardless of starting conformation in the simulations. Moreover, the benzimidazole N3 of **6b** only just overlapped with the edge of Arg45. The carboxylate moiety formed a water-mediated hydrogen bond with the backbone N–H of Gly142 (Figure 4) similar to what was observed in the galectin-8N-**6a** complex (Figure 2A). The benzimidazole of **6a** was during the simulations rotated 180 degrees relative to the benzimidazole of **6b**, placing the carboxylate similar to the position in the X-ray structure and to the carboxylate of **6b**. Consequently, the **6a** benzimidazole N3 hydrogen bound to Gln47 as well as formed intermittent polar interactions with Arg59, during the MD simulation, again reflecting the X-ray structure. The methyl of **6a** stacked onto Arg45, shielding the planar surface of the guanidinium side chain from bulk water. Altogether, the MD essentially reproduced the X-ray structure of **6a** bound to galectin-8N (Figure 2A). The key observation for compound **6b** is that if all three **6a**-**6c** position the carboxylate close to Gly142, then the hydrogen bonding network of Arg59 and Gln47 with a benzimidazole nitrogen lone pairs disrupted only in **6b**.

### Cytotoxicity Evaluation

We have investigated the cytotoxicity of the compounds **1**, **6a**, and **6c** via a (3-(4,5-dimethylthiazol-2-yl)-5-(3- carboxymethoxyphenyl)-2-(4-sulfophenyl)-2*H*-tetrazolium) (MTS) assay in K562 and MDA-MB-231 cancer cell lines to establish the direct antitumor activity of compounds, as well as human peripheral blood mononuclear cells (PBMCs), to evaluate their safety. None of the compounds decreased the viability of cancer cell lines at concentrations ranging from 1 to 100 μM, thus demonstrating a lack of antitumor activity. However, the lack of direct cytotoxicity of compounds **1**, **6a**, and **6c** in PBMCs at concentrations up to 100 μM also renders these compounds suitable as tool compounds to study the biological roles of galectin-8 on the selected cell lines as well as provides a good starting point for the design and synthesis of potent and selective galectin-8N inhibitors.

### Assessment of Cytokine Secretion Profile

**Figure 5:**
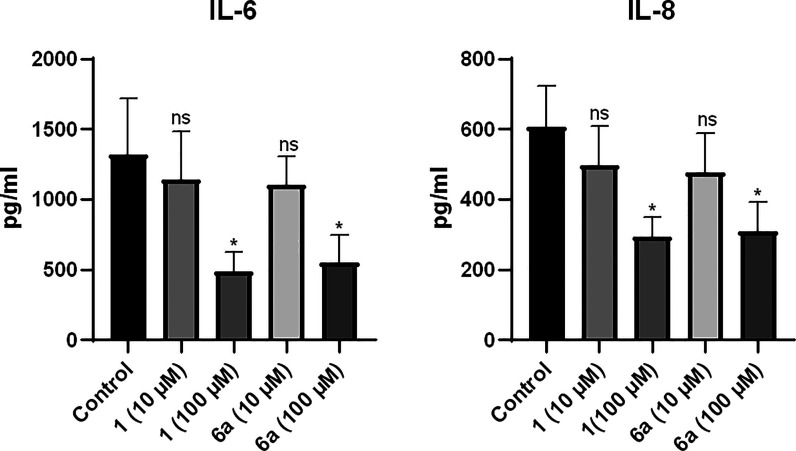
Effect of compounds **1** and **6a** on the secretion of IL-6 and IL-8 expressed in pg/mL. The effects were measured after incubating the cells with the compounds at 10 and 100 μM. Compounds **1** and **6a** reduce the secretion of IL-6 and IL-8 in a dose-dependent manner at 100 and 10 μM. Results shown are means ± SEM of three independent experiments. ns, not significant (*p* > 0.05); *p < 0.05 versus untreated controls.

Triple-negative breast cancer accounts for 15–20% of all breast cancers, with an increased risk of metastasis and a high mortality rate.^33,34^ It is characterized by the absence of estrogen receptor, progesterone receptor, and human epidermal growth factor receptor 2, making it resistant to the clinically available medications for breast cancer.^34^ Importantly, galectin-8 is upregulated in several tumor cells including the triple-negative breast cancer cells MDA-MB-231.^35,36^ A recent study has shown that knocking down galectin-8 in MDA-MB-231 cells prevents cell–cell adhesion while knocking down galectin-8 and its glycosylated ligand activated leukocyte cell adhesion molecule (ALCAM) synergistically delays the tumor growth *in vivo*.^35^ It has been established that galectin-8 upregulates the expression of the proinflammatory cytokines in different cell lines, including osteoblasts, E0771 breast cancer cells, and D122-Luc Lewis lung carcinoma cells in mice as well as vascular endothelial cells.^36,37^ A recent study has shown that treatment of SUM159 breast cancer cells with exogenous galectin-8 stimulated the secretion of IL-6, IL-8 and IL-1β, while cotreatment with galectin-8 antagonists blocked this effect.^18^ Since MDA-MB-231 cells express galectin-8 endogenously, we reasoned that inhibition of endogenous galectin-8 might reduce proinflammatory cytokine secretion in these cells. To this end, we treated MDA-MB-231 cells with compounds **6a** and **1** at two different concentrations (10 and 100 μM). Both compounds markedly decreased the secretion of IL-6 and IL-8 in a dose-dependent manner compared to the untreated cells. At 100 μM, both compounds diminished the secretion of IL-6 by about 65% while reducing the secretion of IL-8 by about 55%. Both compounds were still active at 10 μM, resulting in about 15% reduction in IL-6 secretion and 20% reduction in IL-8 secretion, albeit the effect was not significant (Figure 5). The observed effect is not a result of direct cytotoxicity, as both compounds had no effect on the viability of the cells at the tested concentrations. While the affinity of compound **1** for galectin-8N is about 25-fold higher than that of compound **6a**, their effects on the cytokines secretion at the tested concentrations are similar. On the other hand, compound **6a** binds galectin-8N with higher selectivity compared to compound **1**. Since MDA-MB-231 cells express galectins-1, -3, and -9, compound **1** could potentially bind these intracellular galectins, reducing its available concentration at the binding site of galectin-8N and/or resulting in antagonizing effects on the secretion of cytokines. Moreover, different time-dependent effects could also be ascribed to a different cellular uptake of the compounds. The possibility that both compounds affect cytokine secretion via binding another target with similar *K*_d_ values also must be taken into consideration. This necessitates further investigation of the underlying molecular mechanism and the involvement of galectin-8 in cytokine secretion from MDA-MB-231 cells.

It should be noted that IL-6 and IL-8 play important roles in breast cancer pathophysiology. For example, Wang et al. have shown that IL-6 protects MDA-MB-231 cells from the cytotoxicity and apoptosis induced by chemotherapeutic agents such as doxorubicin and paclitaxel via increased expression of HIF-α.^38^ Similarly, IL-8 is overexpressed in all ER-negative breast cancer cells including MDA-MB-231. Recent studies have shown that IL-8 promotes the migration and metastasis of MDA-MB-231 cells through the induction of the extracellular traps formation, as well as the activation of PI3K-Akt signaling pathway and epithelial–mesenchymal transition.^39^ A previous study has also shown that inhibiting both IL-6 and IL-8 in MDA-MB-231 cells inhibits cell viability, colony formation, as well as cell migration.^40^ Therefore, the inhibitory effects of compounds **1** and **6a** are of significant importance since treatment for the triple-negative breast cancer is limited to cytotoxic agents with limited durable response rates due to chemoresistance that accounts for 90% of drug failures.

## Conclusion

In conclusion, we have designed and synthesized d-galactal derivatives carrying benzimidazolyl- and quinolyl-methyl moieties at O3 that display higher affinity and selectivity for galectin-8N compared to the galactose derivatives. Compound **6a** is the most selective galectin-8N ligand to date with 15-fold selectivity over galectin-3. The quantum mechanical calculations have revealed that the interaction of the LUMO of Arg45 with the HOMO of O4 and the HOMO of the olefin in the galactal ring are responsible for the high affinity and selectivity of the compounds for galectin-8N. Compounds **1**, **6a,** and **6c** were directly cytotoxic to neither cancer cell lines nor healthy cells. Finally, compounds **1** and **6a** have also reduced the secretion of IL-6 and IL-8 in MDA-MB-231 cells in a dose-dependent manner, making them promising starting points toward galectin-8N-inhibitory lead compounds.

## Supporting Information Available

Experimental procedures and physical data for new compounds, procedures for X-ray crystallography and atomic structure determination, biological assay conditions, and molecular modeling procedures (PDF)

## Notes

The crystal structures and the diffraction data have been deposited in the Protein Data Bank under accession IDs 7P1M (**6a** complex) and 7P11 (**9** complex).

## Abbreviations

CRD: carbohydrate recognition domain
N: N-terminal domain
C: C-terminal domain
VEGF-C: vascular endothelial growth factor C
MW: microwave
SEM: standard error of the mean
MD: molecular dynamics
ns: nanosecond
HOMO: highest occupied molecular orbital
LUMO: lowest unoccupied molecular orbital
DMF: *N*,*N*-dimethylformamide
HPLC: high performance liquid chromatography
DMSO: dimethyl sulfoxide
TCEP: (tris(2-carboxyethyl)phosphine)
Tris: (tris(2 carboxyethyl)phosphine)
PEG: polyethylene glycol
MME: monomethyl ethers
PDB: protein data bank
DIPEA: *N*,*N*-diisopropylethylamine
MTS: (3-(4,5-dimethylthiazol-2-yl)-5-(3-carboxymethoxyphenyl)-2-(4-sulfophenyl)-2*H*-tetrazolium)
PBMC: peripheral blood mononuclear cell.
