# Supplementary material for: Structure-Guided Design of d-Galactal Derivatives with High Affinity and Selectivity for the Galectin-8 N-Terminal Domain
Source: ACS Med Chem Lett. 2021 Nov 2;12(11):1745–52. doi: 10.1021/acsmedchemlett.1c00371 (PMC8592027; doi:10.1021/acsmedchemlett.1c00371)

Structure-Guided Design of D-Galactal Derivatives with High Affinity and Selectivity for Galectin-8 N-terminal Domain

Mujtaba Hassan,[a, b] Floriane Baussière,[a] Samo Guzelj,[b] Anders P. Sundin,[a] Maria Håkansson,[c] Rebeka Kovačič,[c] Veronika Klančič,[b] Hakon Leffler,[d] Tihomir Tomašič,[b] Marko Anderluh,[b] Žiga Jakopin,[b, *] Ulf J. Nilsson[a, *]

[a] Centre for Analysis and Synthesis, Department of Chemistry, Lund University, Box 124, SE-221 00 Lund, Sweden
[b] University of Ljubljana, Department of Medicinal Chemistry, Faculty of Pharmacy, Aškerčeva 7, 1000 Ljubljana, Slovenia.
[c] SARomics Biostructures AB, Medicon Village, SE-223 63 Lund, Sweden
[d] Department of Laboratory Medicine, Section MIG, Lund University BMC-C1228b, Klinikgatan 28, 221 84 Lund, Sweden
* Corresponding author

Table of Contents

1. Competitive fluorescence polarization experiment S2
2. X-ray data collection and atomic structure determination S2
3. Chemistry S3
4. Biology S6
5. Molecular modelling S9
6. NMR spectra of the synthesized compounds S10

1. Competitive fluorescence polarization experiments:

Human galectins -1, -3, -4N, -4C, -7, -8N, -9N, and -9C were expressed and purified as previously described [30,41]. Fluorescence polarisation experiments were performed using the PHERAstar FS plate reader (software version 2.10 R3), and the fluorescence anisotropy of fluorescein tagged probes was measured by excitation at 485 nm and emission at 520 nm. The specific conditions for galectins -1, -3, -4N, -4C, -7, -8N, -9N, and -9C were kept as reported[30,41,42]. The synthesised compounds were dissolved in pure DMSO at 20 mM concentration and diluted in PBS to 3-6 different concentrations, and each concentration was tested in duplicate. The highest inhibitor concentrations tested were 1.5 mM. The average values of K_d and SEM were calculated from 4 to 8 duplicate measurements, showing 10-90% inhibition.

2. X-ray data collection and atomic structure determination:

Human galectin-8N was crystallized as described previously.[20] In brief 12 mg/ml galectin-8N in 10 mM lactose, 10 mM Tris/HCl pH 8.0, 1 mM TCEP and 150 mM sodium chloride was mixed with 25 % (w/v) PEG 2000 monomethylether (PEG 2000 MME) and seed solution from crystals grown at the same condition (1.5 mL protein + 1.25 mL reservoir + 0.25 mL seed solution in a hanging drop over 1 ml reservoir using a NEXTAL plate). The experiment was setup at room temperature and crystals appeared within a few days. The co-crystals with lactose were used to soak in compound **6a** or **9** by transferring crystals in three steps to different soaking drops. First to a 2 µL drop with glycerol (20% (v/v) glycerol, 25% (w/v PEG 2000 MME, 10 mM Tris/HCl pH 8.0, 50 mM NaCl and 1 mM TCEP), and secondly to a 2 µL drop with 5 mM compound **6a** or **9** (10 mM Tris/HCl pH 8.0, 50 mM NaCl, 5 mM compound, 25% (w/v) PEG 2000 MME and 1 mM TCEP), and thirdly to a second drop with 5 mM compound **6a** or **9** (same composition as before). The incubation times in the first drops were about 10 min minutes and in the third drop about 24 h. Then the crystals were transferred to a cryo solution (10 mM Tris/HCl pH 8.0, 50 mM NaCl, 5 mM compound **6a** or **9** respectively, 25% (w/v) PEG 2000 MME, 20% ethylene glycol (EG) and 1 mM TCEP) and flash-frozen in liquid nitrogen. Data were collected at Diamond Light Source beamline i03 to 1.52 Å for compound **6a** and at Swiss Light Source beamline PXIII to 2.10 Å for compound **9**. The compound **6a** data set was processed using the Xia2 pipeline[43] and Aimless,[44] and the compound **9** data set using XDS[45] and Aimless[44] programs. Both structures were determined with two molecules in the asymmetric unit in space group $P2_12_12_1$. The structures have been refined starting from the 1.35 Å model of galectin-8N with lactose PDB id: 7ALS determined in the same space group. The refinements were made using Refmac5,[46] the model building was made in Coot,[47] and the models have been analysed using Molprobity,[48] (*Table S1* in the supporting information).

Table S1. Data collection and refinement statistics

	Compound **6a** PDB ID: 7P1M	Compound **9** PDB ID: 7P11
Data collection<br>Space group	$P2_12_12_1$	$P2_12_12_1$
Cell dimensions<br>a, b, c (Å)	54.4, 61.8, 84.6	54.6, 61.4, 85.2
α, β, γ (°)	90, 90, 90	90, 90, 90
Wavelength (Å)	0.97935	1.00003
Resolution (Å)	49.91 – 1.52<br>(1.55 - 1.52)*	45.99 – 2.10<br>(2.16 – 2.10)*
R_{merge}	0.039 (1.61)	0.144 (1.69)
Mean I/σ(I)	19.1 (1.2)	8.8 (1.1)
Completeness (%)	100.0 (100.0)	99.9 (99.8)
Redundancy	6.6 (6.7)	6.4 (6.3)
Refinement (Å)<br>R_{work}/R_{free}	49.9 – 1.52<br>0.145/0.207	44.99 – 2.10<br>0.205/0.263
No. of atoms	2812	2643
Ligands	two compounds **6a**	two compounds **9**
R.m.s. deviation of bonds (Å)	0.013	0.010
R.m.s. deviation of angles (°)	1.7	1.6
Ramachandran**<br>Favoured regions (%)<br>Allowed regions (%)<br>Outliers (%)	 99.0<br>1.0<br>0.0	 98.0<br>2.0<br>0.0

*Outer resolution shell

** Calculated using Molprobity[48]

3. Chemistry:

General procedure:

All reagents and solvents were dried prior to use according to standard methods and commercial reagents were used without further purification. TLC analysis was performed on precoated Merck silica gel 60 F254 plates using UV light and charring solution (10 mL conc. H_2SO_4/90 mL EtOH). Flash column chromatography was performed on SiO_2 purchased from Aldrich (technical grade, 60 Å pore size, 230–400 mesh, 40–63 µm). Reverse-phase purification was done on Biotage® Isolera flash chromatography system using Biotage® SNAP cartridge (KP-C18-HS 30g), or preparative HPLC on an Agilent 1260 Infinity system with a SymmetryPrep C18, 5 µM, 19 mm × 100 mm column using a gradient (water with 0.1% formic acid and acetonitrile). Monitoring and collection were based on UV−vis absorbance at 210 and 254 nm. ^{1}H, ^{13}C, COSY, and HMQC were recorded with a Bruker Avance II 400 MHz spectrometer (400 Hz for ^{1}H, 100 Hz for ^{13}C) at ambient temperature. Chemical shifts are reported in δ parts per million (ppm), with multiplicity (b = broad, s = singlet, d = doublet, t = triplet, q = quartet, quin = quintet, hept = heptet, m = multiplet, app = apparent), coupling constants (in Hz) and integration. High-resolution mass analyses were performed using a Micromass Q-TOF mass spectrometer (ESI). HPLC analyses were performed on a modular system (1100LC; Agilent Technologies, CA, USA), and Thermo Scientific Dionex UltiMate 3000; (Thermo Fisher Scientific Inc., MA, USA). Method: C18 column (Eclipse Plus; 5 µm, 4.6 mm × 150 mm; Agilent), T = 25 °C; sample 0.2 mg/mL in MeCN; flow rate = 1.0 mL/min; detector λ = 220 nm; mobile phase A (0.1% TFA [v/v] in water), mobile phase B (MeCN). Gradient (for mobile phase B): 0−12 min, 10−90%; 12−14 min, 90%; 14−15 min, 90−10%. Purities of the tested compounds were established to be ≥95%, as determined by HPLC. Analytical data are given if the compound is novel or not fully characterised in the literature.

General method for the synthesis of compounds 5a–5c:

D-galactal (**3**) (1 equiv.) and Bu_2SnO (1.1 equiv.), Bu_4NBr or Bu_4NBI (1.5 equiv.), **4a**–**4c** (1 equiv.), and toluene: acetonitrile (5:1) were added to microwave vial and the reaction was run at 120 °C for 30-90 mins. The volatiles were evaporated, and the crude mixture was purified with flash column chromatography. The resulting compounds were further purified with Biotage® Isolera reverse-phase flash chromatography system or preparative HPLC before testing on mammalian galectins.

3-*O*-[(6-Methoxycarbonyl-1-methyl-1*H*-benzo[d]imidazol-2-yl)-methyl]-D-galactal (**5a**):

Following the general method, the reaction was performed with D-galactal (**3**) (101 mg, 0.69 mmol, 1 equiv.), Bu_2SnO (188 mg, 0.75 mmol, 1.1 equiv.), Bu_4NI (380 mg, 1.02 mmol, 1.5 equiv.), **4a** (170 mg, 0.71 mmol, 1 equiv.) and 6 ml of toluene: acetonitrile (1:5**).** Compound **5a** was obtained as a white amorphous solid in 50% yield (120.3 mg). ^{1}H NMR (400 MHz, MeOD) δ 8.26 (d, *J* = 1.0, 1H, ArH), 7.99 (dd, *J* = 8.5, 1.6 Hz, 1H, ArH), 7.71 (d, *J* = 8.5 Hz, 1H, ArH), 6.41 (dd, *J* = 6.3, 1.8 Hz, 1H, H-1), 4.98 (ABq, *J* = 13.0, 12.2 Hz, 2H, $CH_2C_{10}H_{10}N_2O$), 4.73 (dt, *J* = 6.3, 2.0 Hz, 1H, H-2), 4.35 – 4.29 (m, 1H, H-4), 4.19 (dt, *J* = 4.4, 1.7 Hz, 1H, H-3), 3.99 (s, 3H, OCH_3), 3.97 – 3.92 (m, 4H, H-5, NCH_3), 3.88 – 3.75 (m, 2H, H-6a, H-6b). ^{13}C NMR (101 MHz, MeOD) δ 167.38, 154.42, 144.64, 135.65, 124.89, 123.43, 118.20, 112.00, 99.29, 77.37, 72.55, 62.57, 62.26, 60.82, 56.38, 51.28, 29.47. HRMS calcd for $C_{17}H_{20}N_2O_6$ + H $(M + H)^+$: 349.1391, found: 349.1379.

3-*O*-[(5-Ethoxycarbonyl-1-methyl-1*H*-benzo[d]imidazol-2-yl)-methyl]-D-galactal (**5b**):

Following the general procedure, the reaction was performed with D-galactal (**3**) (250 mg, 1.71 mmol, 1 equiv.), Bu_2SnO (496 mg, 1.88 mmol, 1.1 equiv.), Bu_4NBr (828 mg, 2.57 mmol, 1.5 equiv.), **4b** (436 mg, 1.73 mmol, 1 equiv.) and 12 ml of toluene: acetonitrile (1: 5). Compound **5b** was obtained as a

white amorphous solid in 67% yield (417 mg). ^{1}H NMR (400 MHz, DMSO) δ 8.22 (d, *J* = 1.5 Hz, 1H, ArH), 7.91 (dd, *J* = 8.5, 1.6 Hz, 1H, ArH), 7.69 (d, *J* = 8.6 Hz, 1H, ArH), 6.34 (dd, *J* = 6.2, 1.8 Hz, 1H, H-1), 4.90 (ABq, *J* = 21.7, 12.6 Hz, 2H, $CH_2C_8H_7N_2$), 4.62 (dt, *J* = 6.3, 2.0 Hz, 1H, H-2), 4.33 (q, *J* = 7.1 Hz, 2H, OCH_2CH_3), 4.23 (ddt, *J* = 4.7, 2.7, 1.3 Hz, 1H, H-4), 4.06 (dt, *J* = 3.8, 1.8 Hz, 1H, H-3), 3.89 (s, 3H, NCH_3), 3.80 (t, *J* = 6.3 Hz, 1H, H-5), 3.68 – 3.54 (m, 2H, H-6a, H-6b), 1.35 (t, *J* = 7.1 Hz, 3H, OCH_2CH_3). ^{13}C NMR (101 MHz, DMSO) δ 166.71, 154.31, 144.70, 141.67, 139.81, 123.94 (d, *J* = 4.1 Hz), 121.09 (d, *J* = 9.6 Hz), 110.77, 100.72, 78.03, 72.82, 62.93, 61.72, 60.97, 60.33, 56.86, 30.64, 14.72. HRMS calcd for $C_{18}H_{22}N_2O_6$ + H (M + H)$^+$: 363.1550, found: 363.1533.

3-*O*-[(5-Methoxycarbonyl-1*H*-benzo[d]imidazol-2-yl)-methyl]-D-galactal (**5c**):

Following the general procedure, the reaction was performed with D-galactal (**3**) (321 mg, 2.20 mmol, 1 equiv.), Bu_2SnO (601 mg, 2.42 mmol, 1.1 equiv.), Bu_4NBr (1.06 g, 3.29 mmol, 1.5 equiv.), **4c** (713 mg, 2.20 mmol, 1 equiv.) and 12 ml of toluene: acetonitrile (1:5). Compound **5c** was obtained as a white amorphous solid in 44% yield (303 mg). ^{1}H NMR (400 MHz, MeOD) δ 8.25 (s, 1H, ArH), 7.94 (dd, *J* = 8.5, 1.6 Hz, 1H, ArH), 7.60 (d, *J* = 8.5 Hz, 1H, ArH), 6.43 (dd, *J* = 6.3, 1.8 Hz, 1H, H-1), 4.93 (q, *J* = 13.6, 8.0 Hz, 2H, $CH_2C_9H_7N_2O_2$), 4.39 – 4.32 (m, 1H, H-4), 4.26 – 4.19 (m, 1H, H-3), 3.99 – 3.91 (m, 4H, H-5, OCH_3), 3.90 – 3.78 (m, 2H, H-6a, H-6b). ^{13}C NMR (101 MHz, MeOD) δ 167.58, 154.95, 144.63, 131.20, 129.29, 124.26, 123.66, 99.29, 77.28, 72.83, 63.42, 62.59, 60.86, 51.21, 47.61 (dp, *J* = 42.9, 21.4 Hz). $C_{16}H_{18}N_2O_6$ + H (M + H)$^+$: 335.1237, found: 335.1221.

General procedure for compounds 6a–6c and 9:

To a suspension of compounds **5a**–**5c** (1 equiv.) in EtOH–H_2O (3:1), KOH was added, and the mixture was stirred at 80 °C. The volatiles were evaporated under reduced pressure and the resulting solid was purified with Biotage® Isolera reverse-phase flash chromatography system or preparative HPLC to obtain compounds **6a**–**6c**.

3-*O*-[(6-carboxy-1-methyl-1*H*-benzo[d]imidazol-2-yl)-methyl]-D-galactal (**6a**):

Following the general procedure, the reaction was performed with **5a** (53.7 mg, 0.15 mmol), KOH (34.6 mg, 0.61 mmol, 4 equiv.). Compound **6a** was obtained as an amorphous white solid in 41% yield (21 mg, yield). ^{1}H NMR (400 MHz, MeOD) δ 8.15 (d, *J* = 1.8 Hz, 1H, ArH), 7.97 (dd, *J* = 8.4, 1.5 Hz, 1H, ArH), 7.60 (d, *J* = 8.2 Hz, 1H, ArH), 6.40 (dd, *J* = 6.3, 1.8 Hz, 1H, H-1), 4.95 (ABq, *J* = 14.0, 13.1 Hz, 2H, $CH_2C_{10}H_{10}N_2O_2$), 4.72 (dt, *J* = 6.3, 2.0 Hz, 1H, H-2), 4.33 – 4.26 (m, 1H, H-4), 4.19 (dt, *J* = 4.3, 1.7 Hz, 1H, H-3), 3.98 – 3.92 (m, 4H, H-5, NCH_3), 3.88 – 3.77 (m, 2H, H-6a, H-6b). ^{13}C NMR (101 MHz, MeOD) δ 173.97, 152.64, 144.57, 142.67, 135.49, 133.32, 123.87, 117.12, 111.04, 99.38, 77.39, 72.30, 62.55, 62.25, 60.88, 29.26. HRMS calcd for $C_{16}H_{18}N_2O_6$ + H (M + H)$^+$: 335.1237, found: 335.1222.

3-*O*-[(5-carboxy-1-methyl-1*H*-benzo[d]imidazol-2-yl)-methyl]-D-galactal (**6b**):

Following the general procedure, the reaction was performed with **5b** (42 mg, 0.11 mmol), KOH (26 mg, 0.46 mmol, 4 equiv.). Compound **6b** was obtained as an amorphous white solid in 65% yield (25 mg). ^{1}H NMR (400 MHz, MeOD) δ 8.33 (s, 1H, ArH), 8.03 (d, *J* = 8.4 Hz, 1H, ArH), 7.48 (d, *J* = 8.5 Hz, 1H, ArH), 6.40 (d, *J* = 6.3 Hz, 1H, H-1), 4.93 (q, *J* = 17.1 Hz, 2H, $CH_2C_9H_7N_2O_2$), 4.71 (dd, *J* = 6.3, 1.8 Hz, 1H, H-2), 4.29 (s, 1H 4.29 (d, *J* = 2.4 Hz, 1H, H-4), 4.19 (d, *J* = 4.2 Hz, 1H, H-3), 3.98 – 3.91 (m, 4H, H-5, NCH_3), 3.90 – 3.75 (m, 2H, H-6a, H-6b). ^{13}C NMR (101 MHz, MeOD) δ 174.23, 152.11, 144.57, 140.63, 137.41, 132.70, 124.74, 120.02, 108.42, 99.40, 77.38, 72.25, 62.52, 62.21, 60.91, 47.60, 47.39, 47.18, 46.97. HRMS calcd for $C_{16}H_{18}N_2O_6$ + H (M + H)$^+$: 335.1237, found: 335.1223.

3-*O*-[(5-Carboxy-1*H*-benzo[d]imidazol-2-yl)-methyl]-D-galactal (**6c**):

Following the general procedure, the reaction was performed with **5c** (149 mg, 0.44 mmol), KOH (103 mg, 1.8 mmol, 4 equiv.). Compound **6c** was obtained as an amorphous white solid in 76% yield (108 mg, 76%). ^{1}H NMR (400 MHz, MeOD) δ 8.24 (s, 1H), 7.95 (dd, *J* = 8.4, 1.5 Hz, 1H), 7.52 (d, *J* = 8.5 Hz, 1H), 6.42 (dd, *J* = 6.3, 1.8 Hz, 1H), 4.92 – 4.82 (m, 1H, $CH_2C_8H_5N_2O_2$), 4.80 (dt, *J* = 6.3, 2.0 Hz, 1H, H-2), 4.36 – 4.29 (m, 1H, H-4), 4.24 – 4.18 (m, 1H, H-3), 3.98 – 3.92 (m, 1H, H-5), 3.93 – 3.75 (m, 2H, H-6a, H-6b). ^{13}C NMR (101 MHz, MeOD) δ 174.36, 153.34, 144.57, 132.61, 123.99, 99.39, 77.30, 72.66, 63.44, 62.58, 60.93, 47.41, 47.20, 46.98. HRMS calcd for $C_{15}H_{16}N_2O_6$ + H (M + H)$^+$: 321.1087, found: 321.1096.

3-*O*-[(7-Methoxycarbonyl-quinolin-2-yl)-methyl]-D-galactal (**8**)**:**

D-Galactal (130.7 mg, 0.89 mmol), compound **7** (476.8 mg, 1.70 mmol), Bu_2SnO (23.3 mg, 0.09 mmol), Bu_4NBr (87.3 mg, 0.27 mmol) and DIPEA (0.3 mL, 1.72 mmol) were stirred in a closed flask at 85 °C for 1.5 h. The crude was purified by flash chromatography (DCM/MeOH 25:1). The orange product was recrystallized in MeOH to give compound **8** (155.5 mg, 50 %) as a white solid. The product was further purified by preparative HPLC. ^{1}H NMR (400 MHz, $CDCl_3$): δ 9.42 (d, 1H, *J* 8.9 Hz, Ar*H*), 8.33 (dd, 1H, *J* 7.3 Hz, *J* 1.2 Hz, Ar*H*), 8.30 (d, 1H, *J* 8.5 Hz, Ar*H*), 7.78 (dd, 1H, *J* 8.4 Hz, *J* 7.4 Hz, Ar*H*), 7.51 (d, 1H, *J* 9.0 Hz, Ar*H*), 6.55 (dd, 1H, *J* 6.3 Hz, *J* 1.6 Hz, H-1), 5.17 (d, 1H, *J* 14.8 Hz, $CH_2C_{11}H_8NO_2$), 4.89 (d, 1H, *J* 14.9 Hz, $CH_2C_{11}H_8NO_2$), 4.84 (dt, 1H, *J* 6.2 Hz, *J* 2.1 Hz, H-2), 4.37 (m, 1H, H-3), 4.23 (m, 1H, H-4), 4.13-3.97 (m, 3H, H-5, H-6a, H-6b), 4.04 (s, 3H, OCH_3). ^{13}C NMR (100 MHz, $CDCl_3$): δ 145.41, 135.80, 133.98, 130.94, 128.76, 99.64, 75.82, 74.03, 71.49, 64.52, 63.30, 52.40. HRMS calcd for $C_{18}H_{19}NO_6$ + H (M + H)$^+$: 346.1291, found: 346.1284.

3-*O*-[(7-Carboxy-quinolin-2-yl)-methyl]-D-galactal (**9**):

KOH (29.5 mg, 0.53 mmol) was added to a mixture of compound **8** (83.3 mg, 0.24 mmol) in EtOH/H_2O 3:1 (16 mL). The mixture was stirred at 60 °C for 2h. The solvent was evaporated and the crude was purified by flash chromatography (DCM/MeOH 8:2, few drops Et_3N) to give compound **9** (66.7 mg, 83%) as a white solid. The product was further purified by preparative HPLC. ^{1}H NMR (400 MHz, MeOD) δ 9.36 (d, 1H, *J* 8.9 Hz, Ar*H*), 8.18 (dd, 1H, *J* 7.2 Hz, *J* 1.1 Hz, Ar*H*), 8.16 (d, 1H, *J* 8.4 Hz, Ar*H*), 7.79 (dd, 1H, *J* 8.4 Hz, *J* 7.2 Hz, Ar*H*), 7.69 (d, 1H, *J* 8.9 Hz, Ar*H*), 6.43 (dd, 1H, *J* 6.3 Hz, *J* 1.7 Hz, H-1), 4.94 (d, 2H, *J* 1.9 Hz, $CH_2C_{10}H_6NO_2$), 4.80 (dt, 1H, *J* 6.4 Hz, *J* 2.0 Hz, H-2), 4.37 – 4.33 (m, 1H, H-3), 4.22 (dt, *J* = 4.2, 1.6 Hz, 1H, H-4), 3.99 – 3.94 (m, 1H, H-5), 3.91-3.80 (m, 2H, H-6a, H-6b). ^{13}C NMR (100 MHz, MeOD): δ 171.53, 159.13, 146.88, 144.45, 136.20, 132.28, 130.83, 128.88, 128.68, 125.84, 120.11, 99.85, 77.26, 73.08, 70.68, 62.51, 60.97. HRMS calcd for $C_{17}H_{17}NO_6$ + H (M + H)$^+$: 332.1134, found: 332.1132.

4. Biology:

Cell culture:

Human PBMCs from three healthy and consistent donors were isolated from heparinized blood by density gradient centrifugation with Ficoll-Paque (Pharmacia, Sweden) according to the procedure described in the literature.[49] MDA-MB-231 and HepG2 cells (ATCC) were cultured in DMEM (Sigma, Germany), while PBMCs and K562 cells (ATCC) were cultured in RPMI 1640 (Sigma, Germany). Both growth media were supplemented with 100 U/mL penicillin (Sigma-Aldrich, St. Louis/ MO, USA), 100 mg/mL streptomycin (Sigma-Aldrich, St. Louis/MO, USA), 2 mM L-glutamine (Sigma-Aldrich, St. Louis/MO, USA), and 10% heat-inactivated fetal bovine serum (Gibco, USA). Cells were cultured in a humid atmosphere at 37 ◦C and 5% CO_2.

Metabolic activity assay:

The metabolic activities of the cell lines were determined using the CellTiter 96 Aqueous One Solution cell proliferation assay (MTS Reagents, Promega, Madison, WI, USA), according to the manufacturer instructions. Briefly, the cells were treated with the selected compounds in 0.1% dimethyl sulfoxide (DMSO; vehicle), 0.1% DMSO (control vehicle), or medium (control) and seeded at a cell density of 5,500 cells/well (MDA-MB-231 cells) or 4 x 10^4 cells/well (K562 cells), 7000 cells/well (HepG2 cells) or 1 x 10^5 cells/well (human PBMCs) in duplicates (MDA-MB-231, K562, and MDA-MB-231 cells) or triplicates for HepG2 cells (100 µL), in sterile 96-well plates. For the MDA-MB-231 and HepG2, the cells were left to adhere overnight before adding the compounds. The plates were then incubated for 24 hours (48 hours for HepG2), after which 15 µL of MTS reagent was added to each well. After 30-90 minute of incubation, the absorbance was measured at 492 nm using a microplate reader (Synergy HTX Multi-Mode; BioTek Instruments, Inc., VT, USA). These data are presented as percentages relative to the control cells.

Multiplexed cytokine assay:

MDA-MB-231 cells were seeded in 96-well flat-bottom plates at a density of 2 x 10^4 cells/well in 100 µL of medium and allowed to adhere for 24 hours at 37 °C and 5% CO_2. The medium was then replaced by a fresh medium containing the desired concentrations of compounds **1** and 6a, after which the cells were cells incubated again for 24 hours at 37 °C and 5% CO_2. Cell-free supernatants were collected and stored at – 80 °C before the analysis. The cytokines secretion was assessed by BD Cytometric Bead Array Human inflammatory cytokine Kit (content: IL-1, IL-6, IL-8, IL-10, IL-12p). The flow cytometric analyses were performed on an Attune NxT flow cytometer (Thermo Fisher Scientific, Waltham, MA, USA). Standard curves were generated using recombinant cytokines provided in the kit, and the data were analyzed with FlowJo software (Tree Star, Inc., Ashland, OR). The results are expressed in pg/mL. Data are represented as means ± standard deviation (SD) from three representative independent experiments.

Statistical analysis:

All experiments were performed at least twice, with average values expressed as means ± SD. All data were analyzed with the statistical software GraphPad Prism 8.0 (GraphPad Software, Inc., CA, USA). by one-way ANOVA followed with Dunnett's multiple comparison test. Differences were considered non-significant for $p > 0.05$, and significant (*) for $p < 0.05$.

Metabolic activity assay figures:

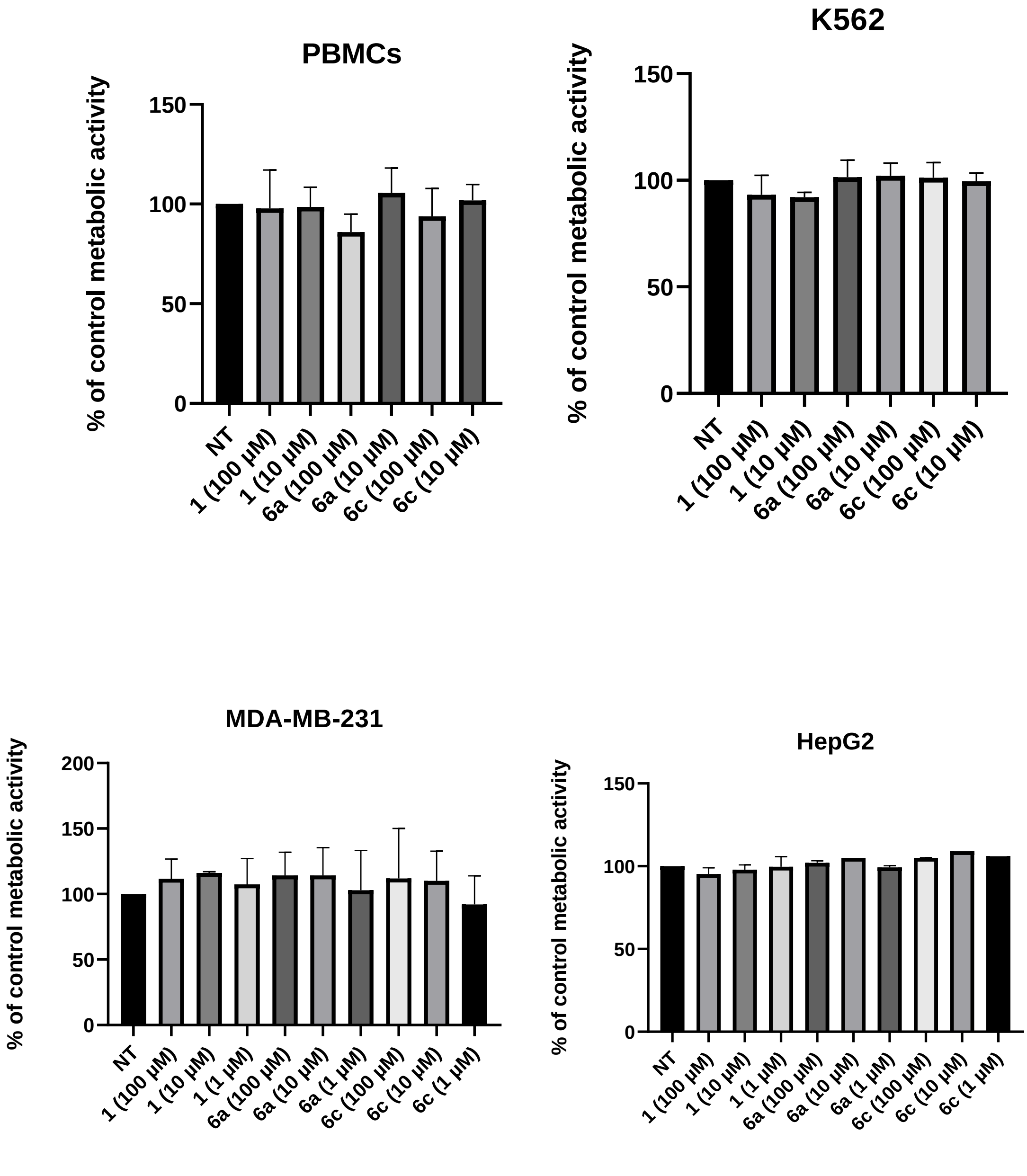

Figure S1: MTS cell proliferation assay of compounds 1, 6a and 6c on human PBMCs, as well as the cancer cell lines K562, MDA-MB-231 and HepG2. . The results are shown relative to that of the untreated controls (NT = 0.1 % DMSO). Data are represented as means ± standard deviation of three independent experiments for human PBMCs, MDA-MB-231, and K562, and two independent experiments for HepG2 cells.

5. Molecular modelling:

Molecular dynamics simulations

Molecular dynamics simulations were performed using the OPLS3 force field in Desmond implemented in Schrödinger Release 2021-1, using default settings except for the length of the simulation, and the use of light harmonic constraints (1 kcal mol^{-1} $Å^{-2}$) on all stranded backbone atoms and the galactose O4 atom. To keep galectin-8N simulations stable, the coulombic cut-off radius had to be increased from the default 9 Å to 10 Å. The galectin-8N-**6a** complex structure with the pdb id 7P1M was used for a 300 ns simulation. In case of simulations with **3** (300 ns) and **6b** (200 ns), the ligand structures were built from the galectin-8N-**6a** complex (pdb id 7P1M).

Quantum mechanical calculations

Snapshots from the MD simulations with **3** (202 ns) and **6a** (265 ns) were used to contruct models for QM calculations. The galectin-8N protein was simplified to residues Arg45, Arg69, Asn67, Val77, Trp86, Asp49, and His65. Single point energy calculations at the HF/4-21G* level were performed and the molecular orbitals from HOMO -30 to LUMO +30 were calculated with Jaguar implemented in the Schrödinger release 2021-1. Plotting HOMO-6, HOMO-27 and LUMO+30.

6. NMR spectra of compouds 5a–5c, 6a–6c, 8, and 9:

3-*O*-[(6-Methoxycarbonyl)-1-methyl-1*H*-benzo[d]imidazol-2-yl)-methyl]-D-galactal (**5a**):

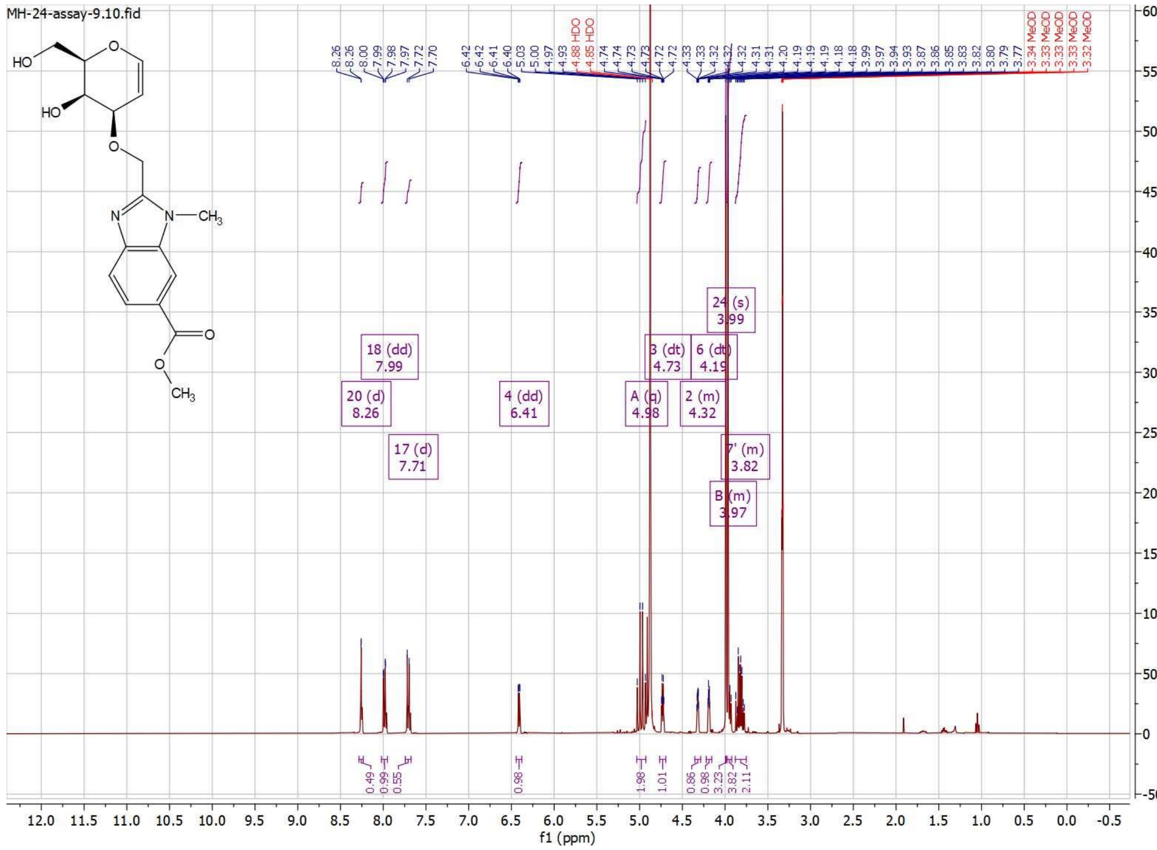

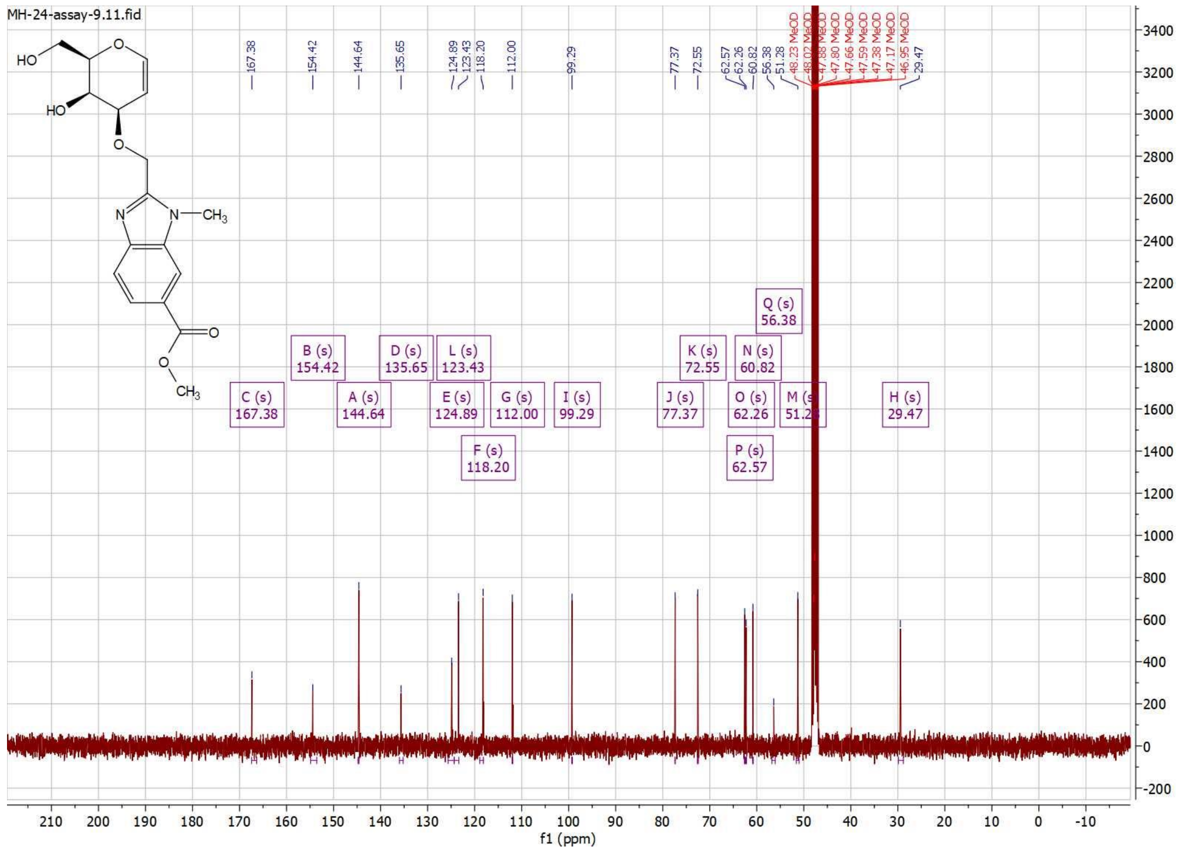

3-*O*-[(5-Ethoxycarbonyl)-1-methyl-1*H*-benzo[d]imidazol-2-yl)-methyl]-D-galactal (**5b**):

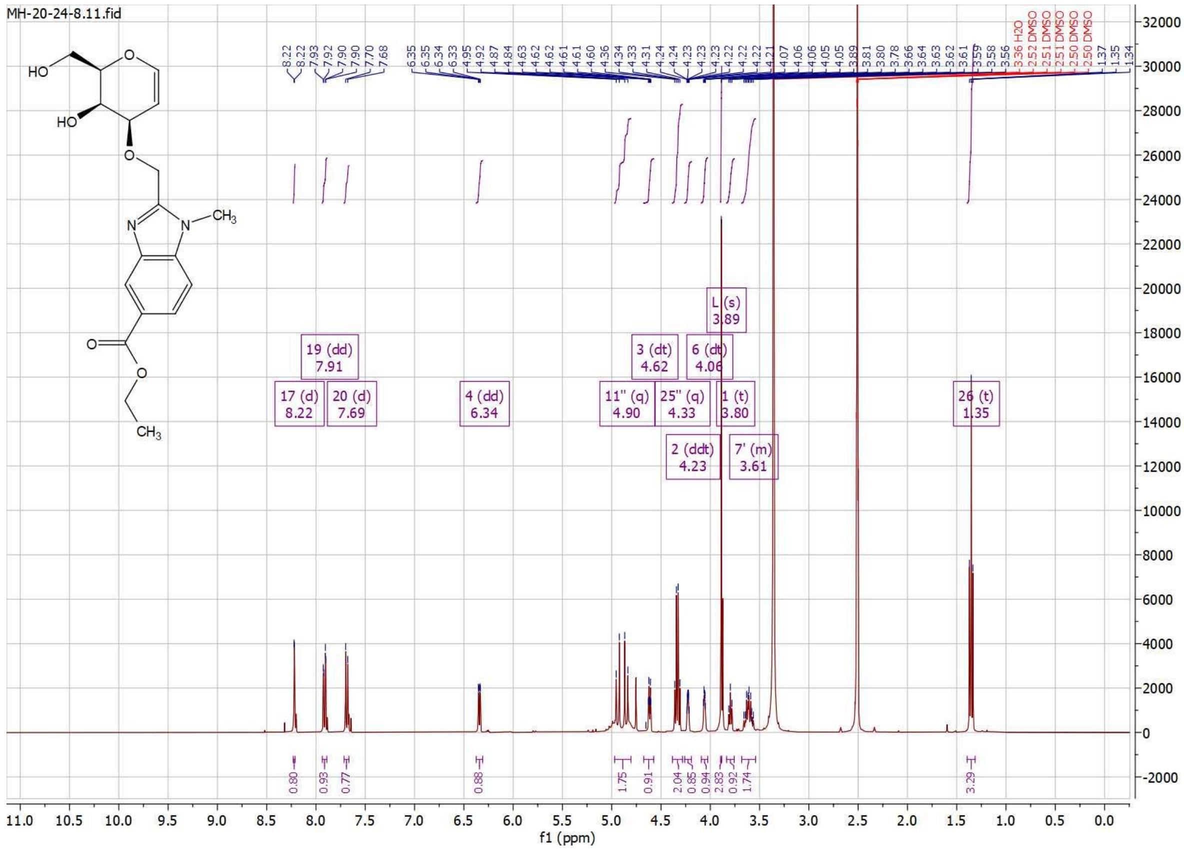

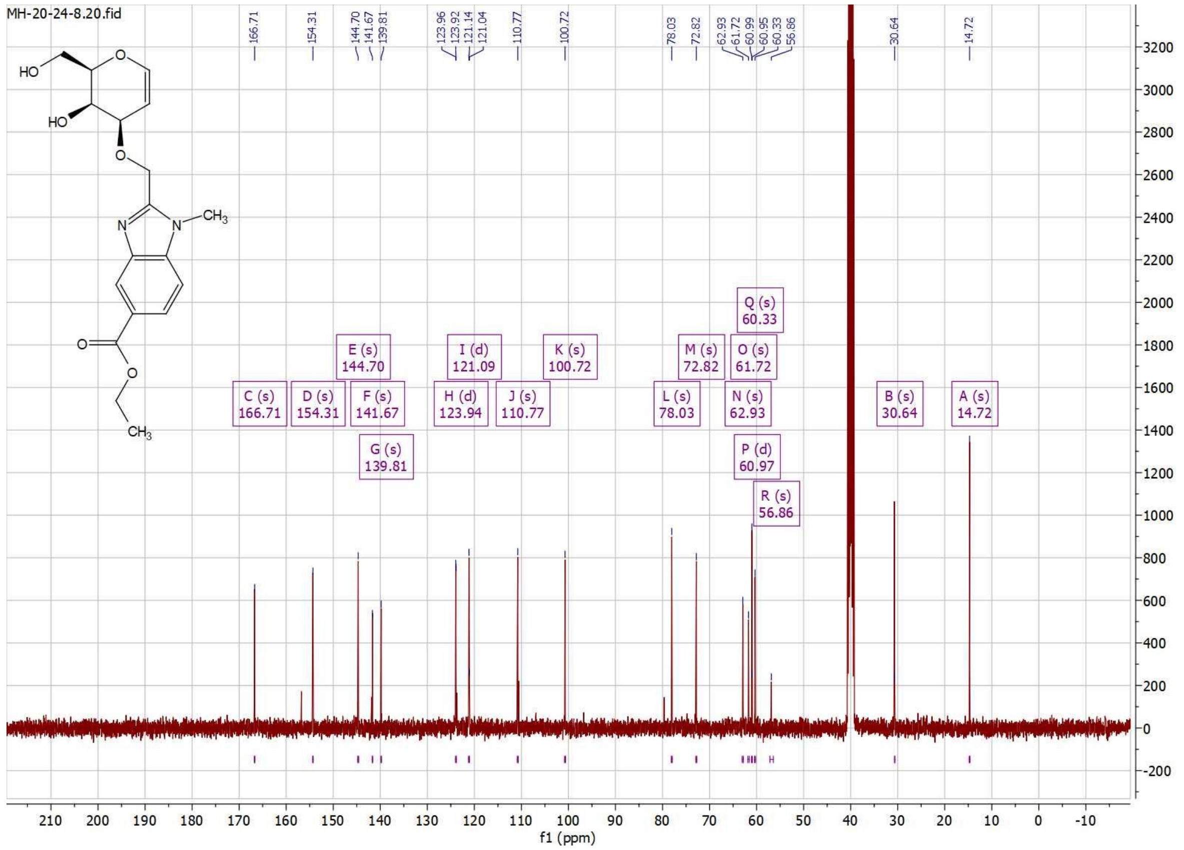

3-*O*-[(5-Methoxycarbonyl)-1*H*-benzo[d]imidazol-2-yl)-methyl]-D-galactal (**5c**):

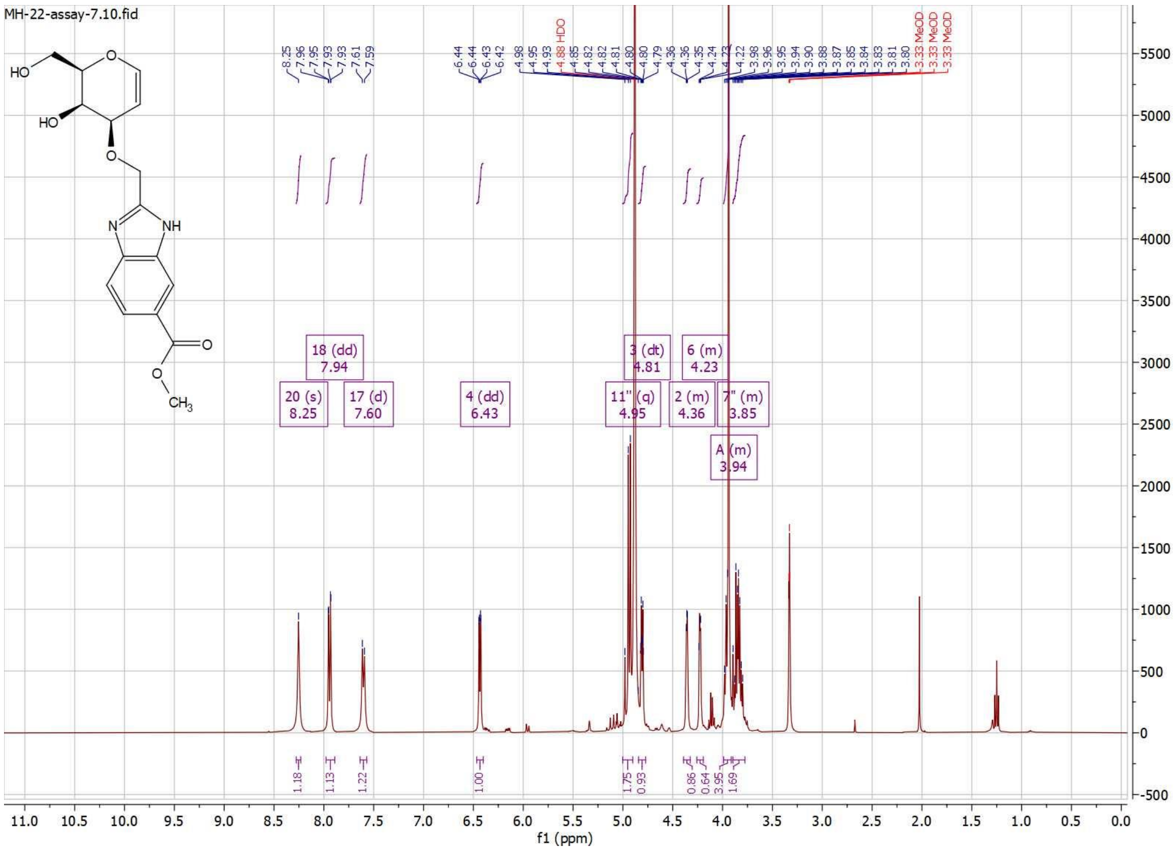

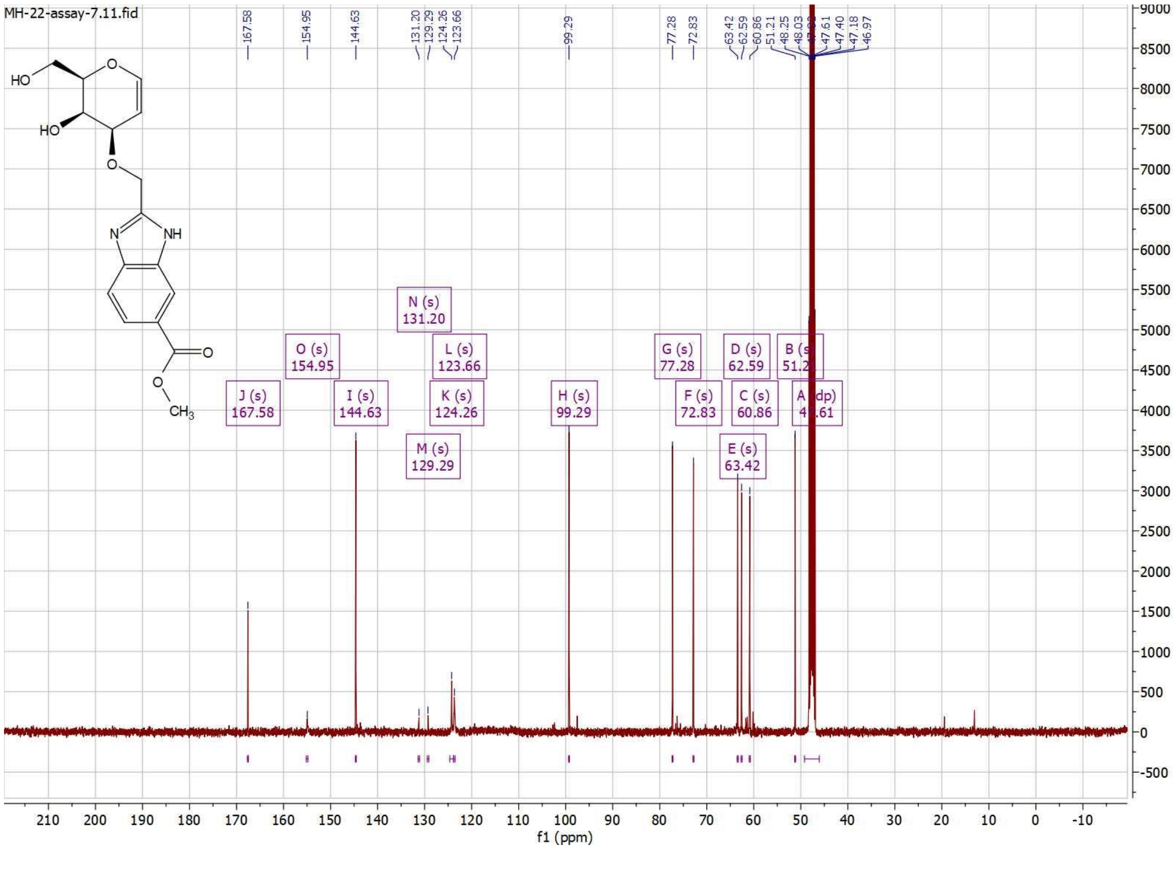

3-*O*-[(6-Carboxy)1-methyl-1*H*-benzo[d]imidazol-2-yl)-methyl]-D-galactal (**6a**):

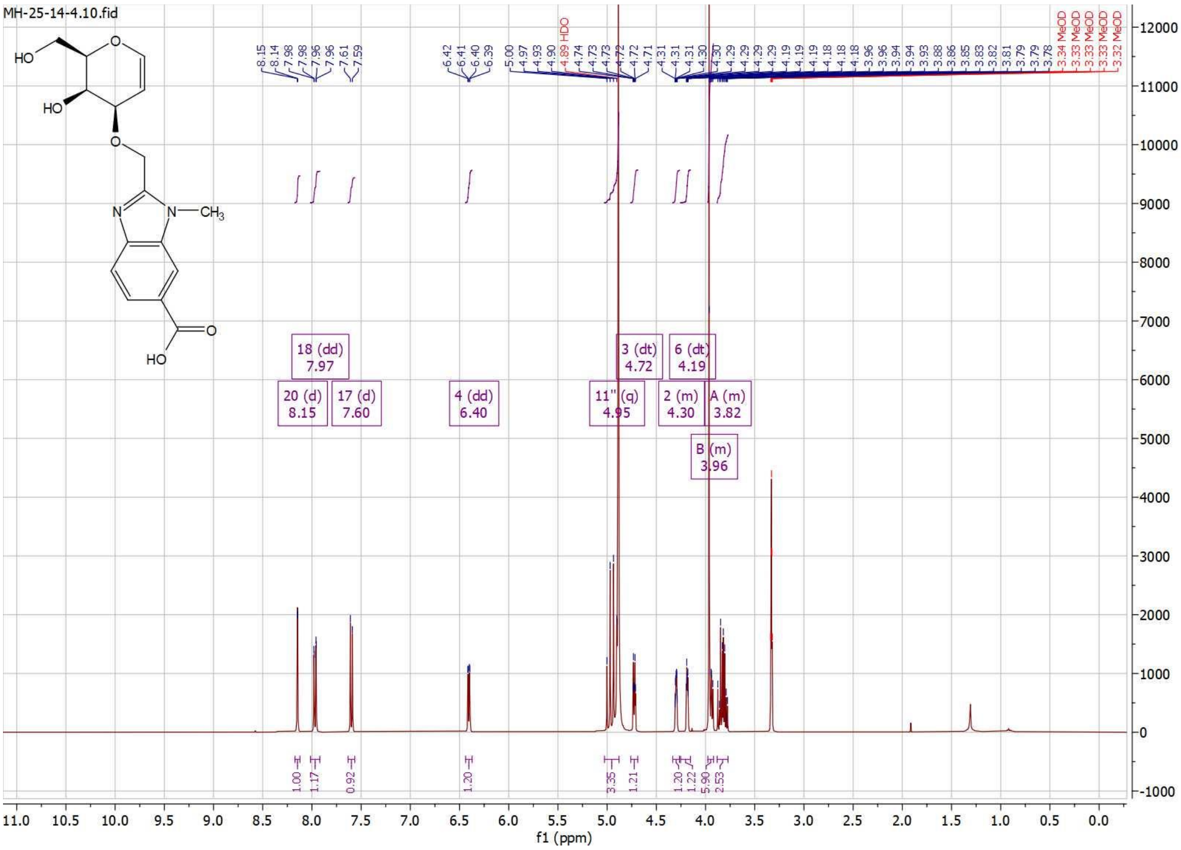

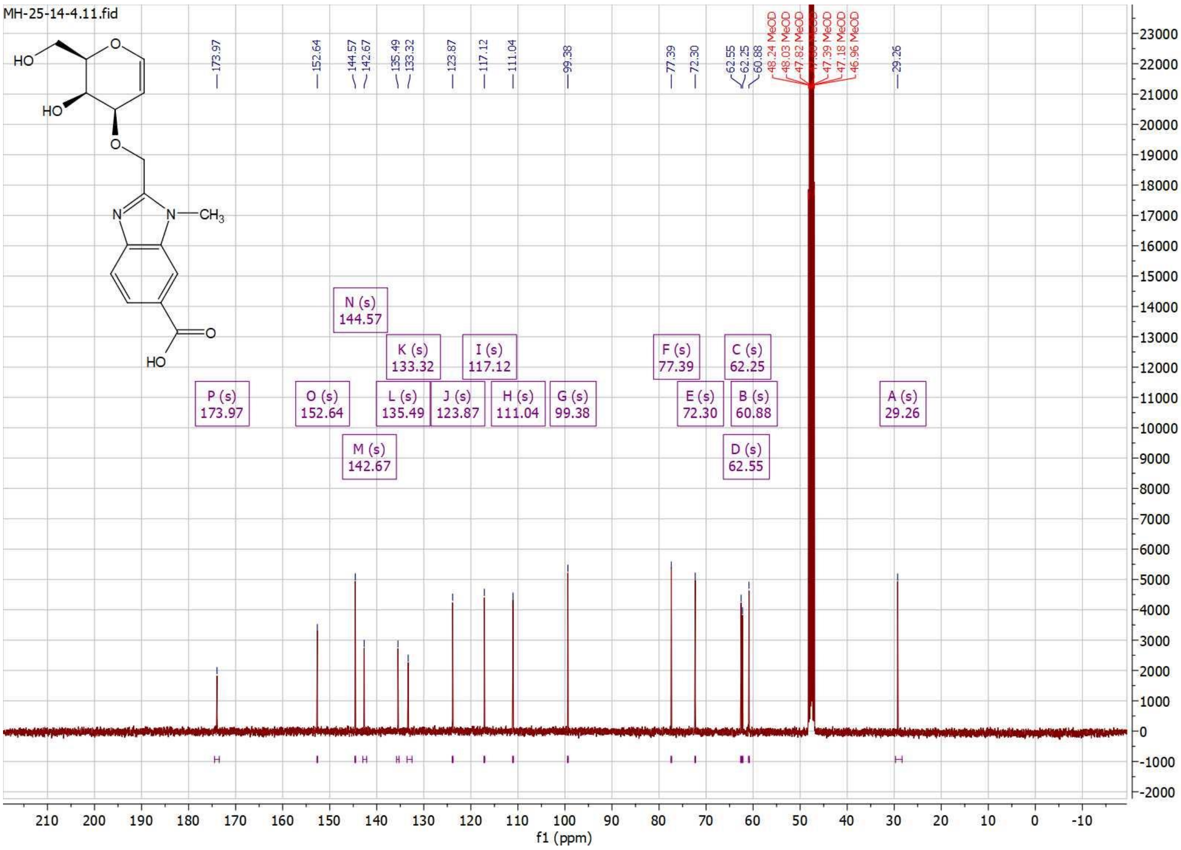

3-*O*-[(5-Carboxy)1-methyl-1*H*-benzo[d]imidazol-2-yl)-methyl]-D-galactal (**6b**):

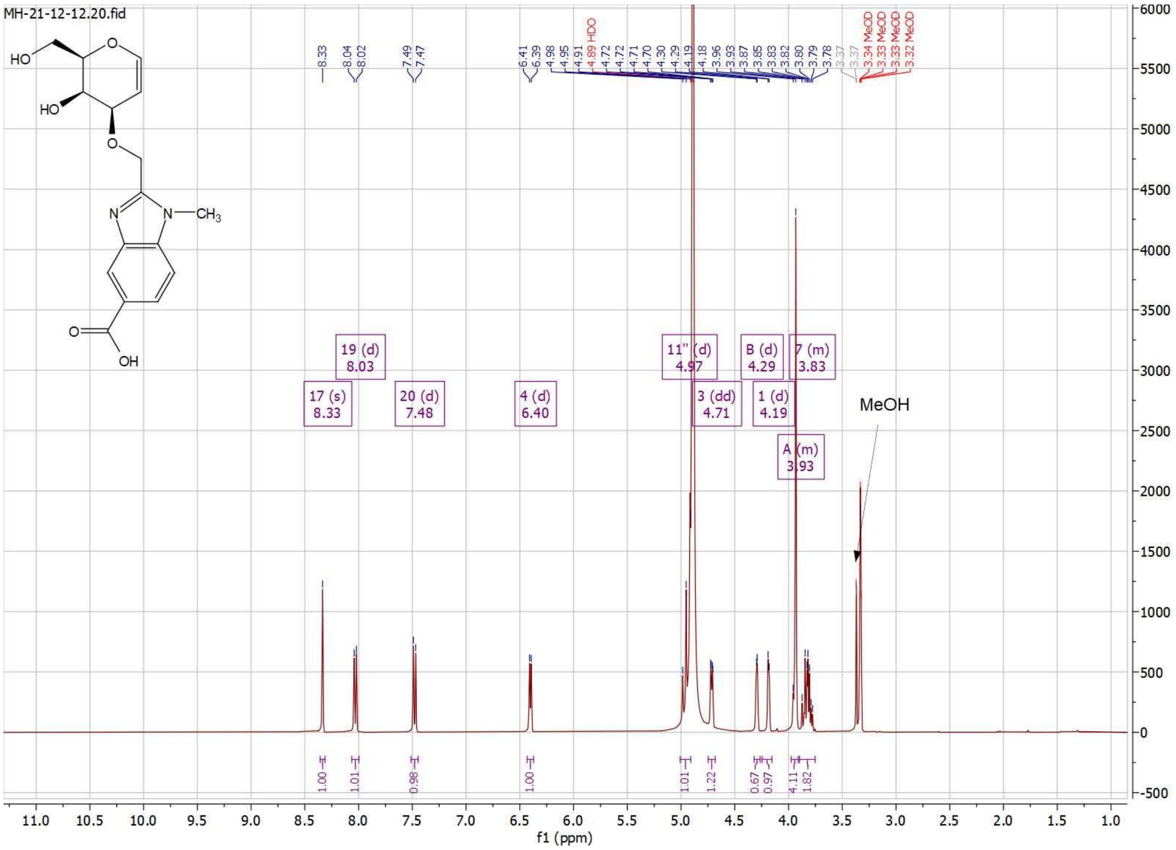

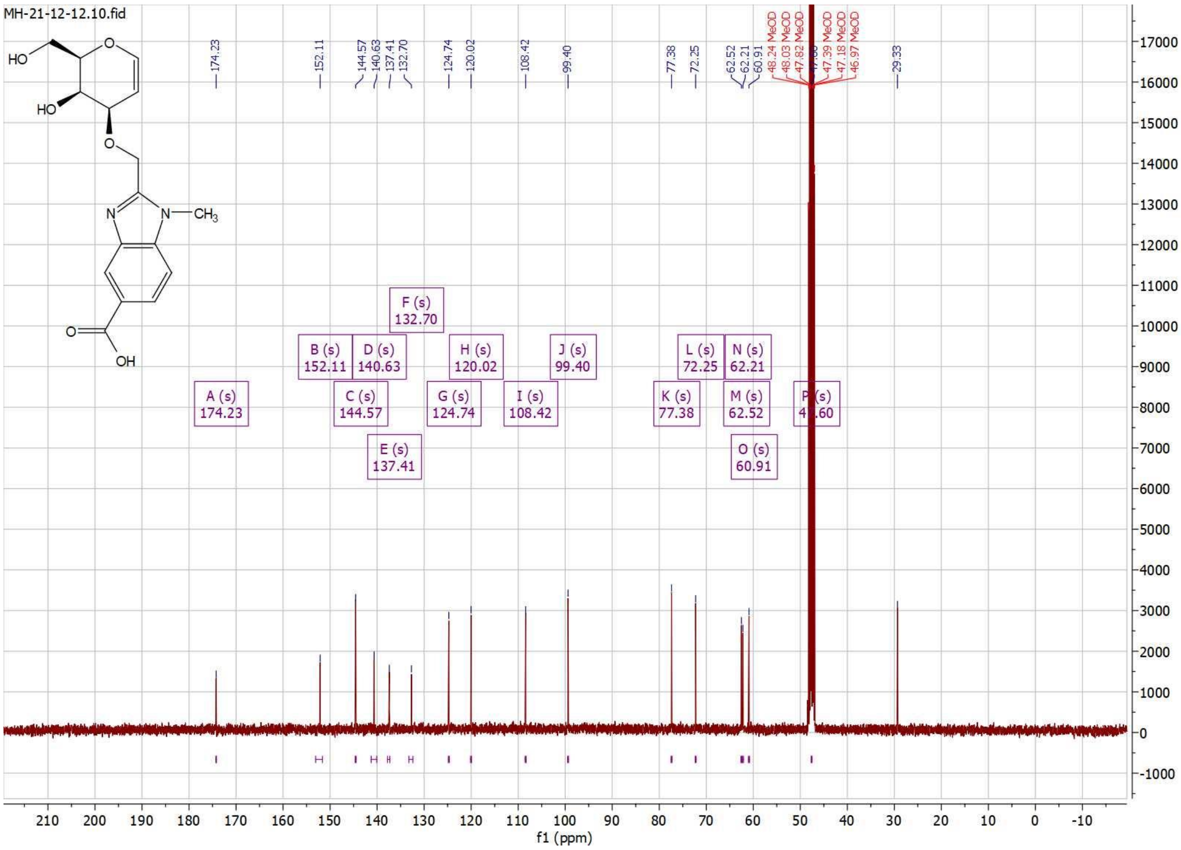

3-*O*-[5-Carboxy-1*H*-benzo[d]imidazol-2-yl)-methyl]-D-galactal (**6c**):

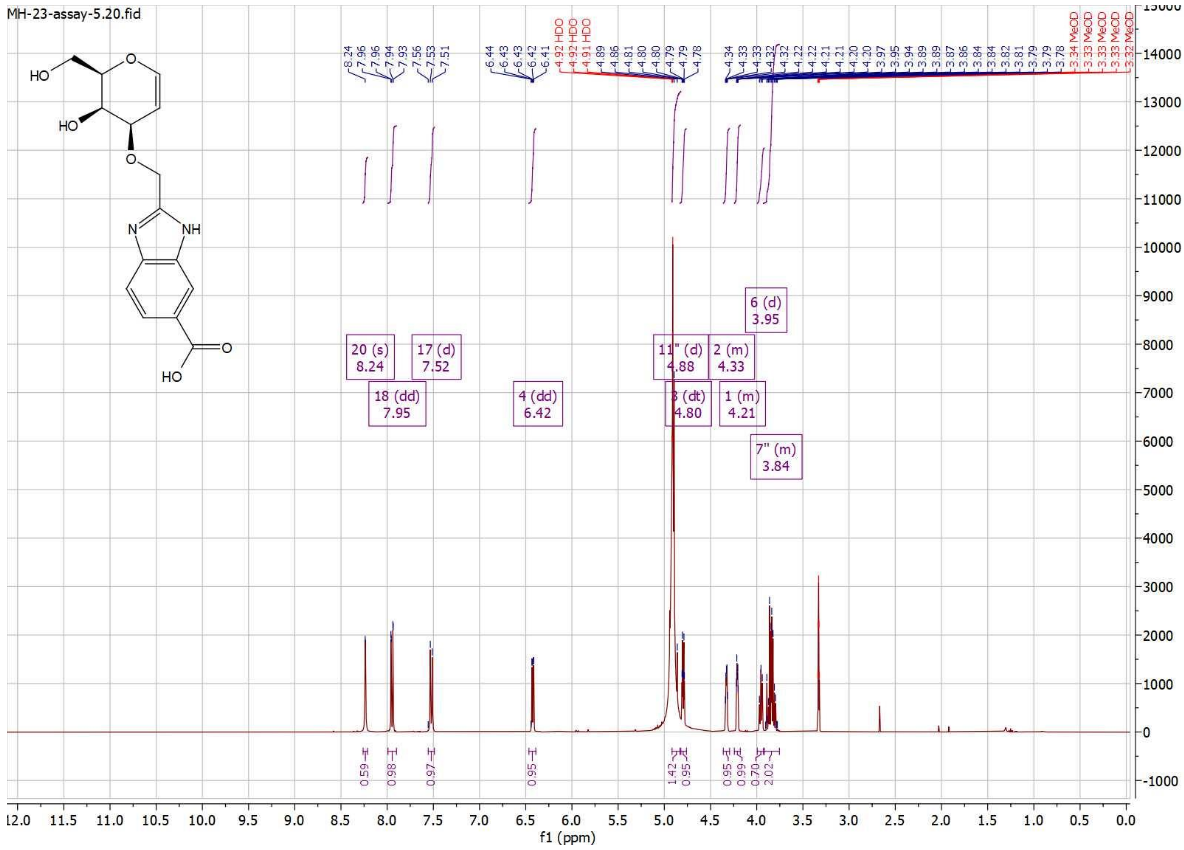

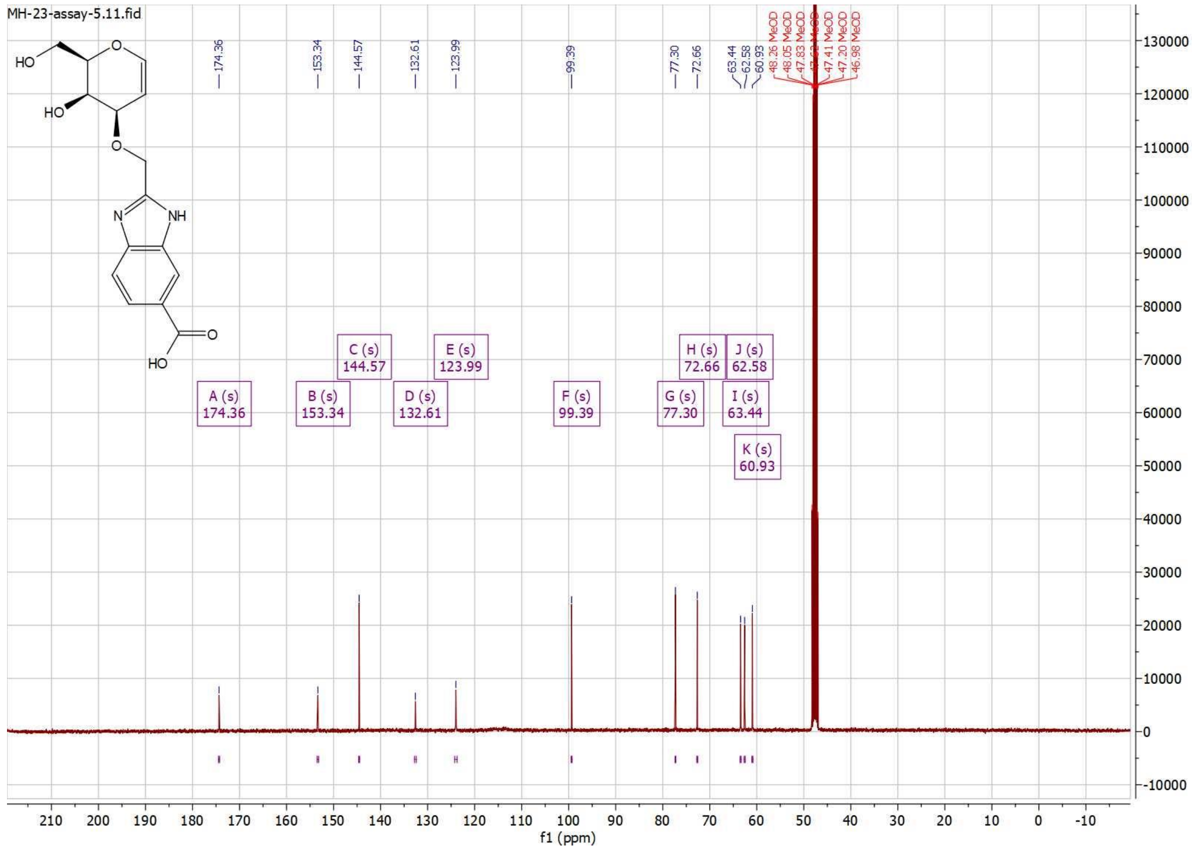

3-O-[(7-Methoxycarbonyl-quinolin-2-yl)-methyl]-D-galactal (**8**):

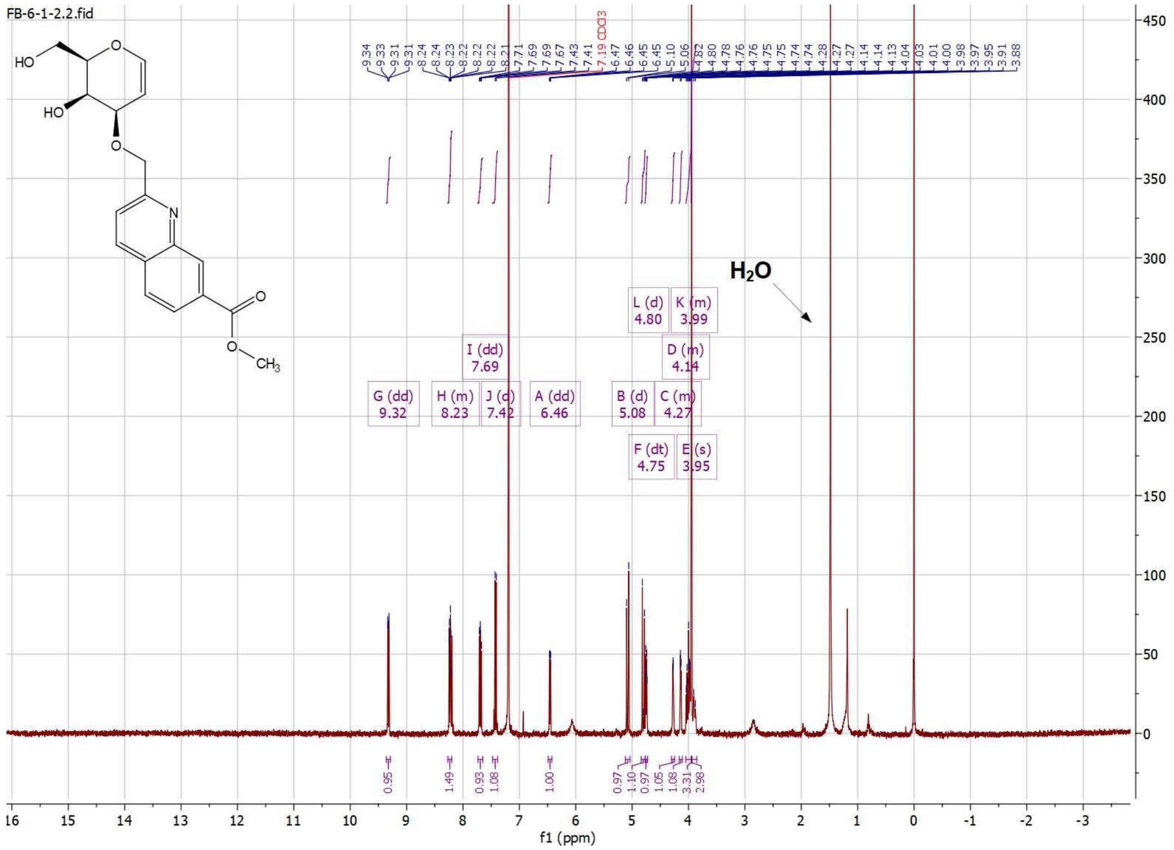

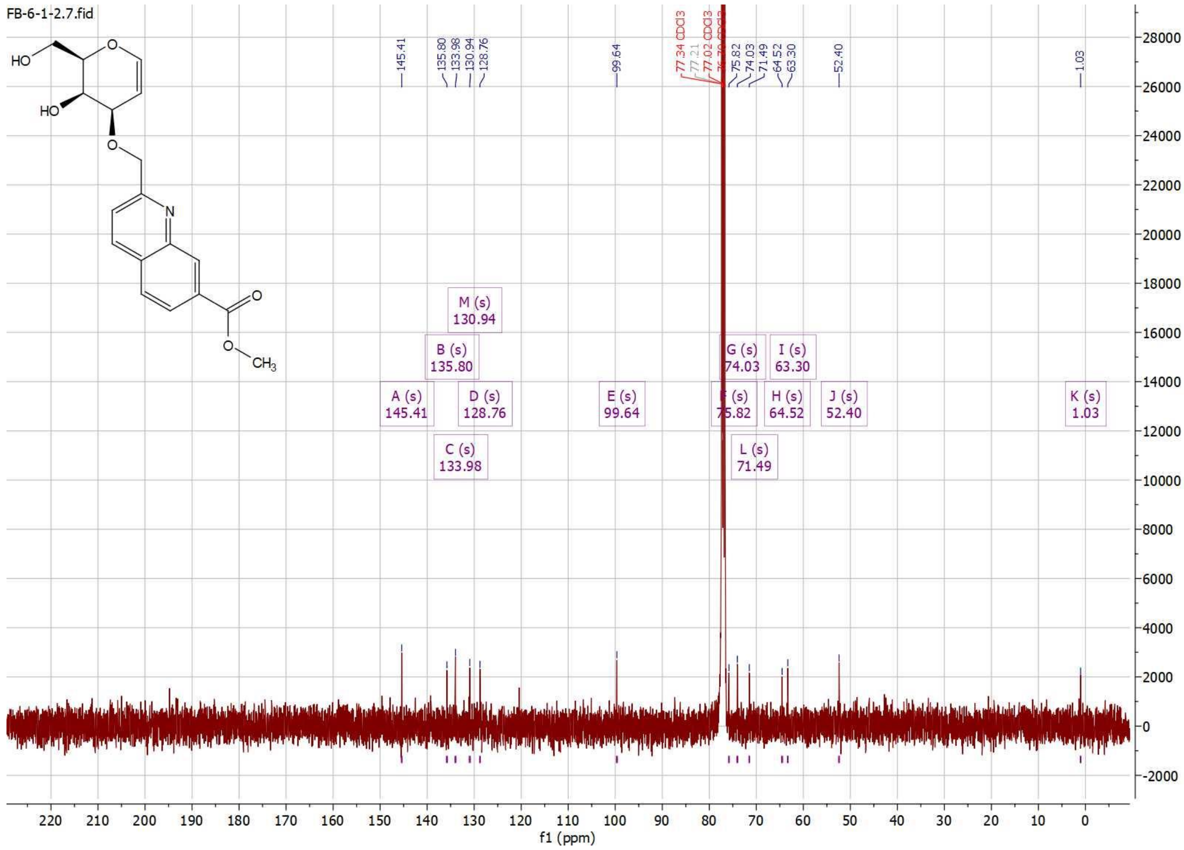

3-O-[(7-Carboxy-quinolin-2-yl)-methyl]-D-galactal (**9**):

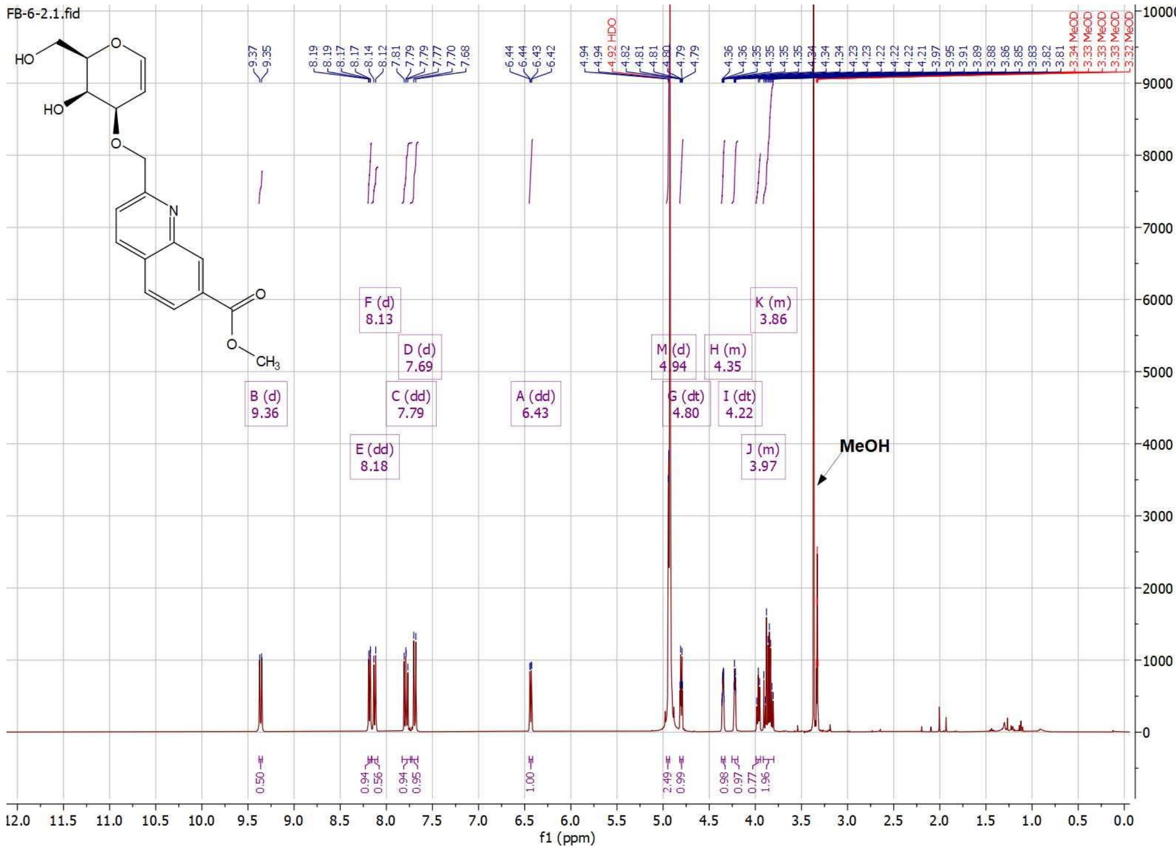

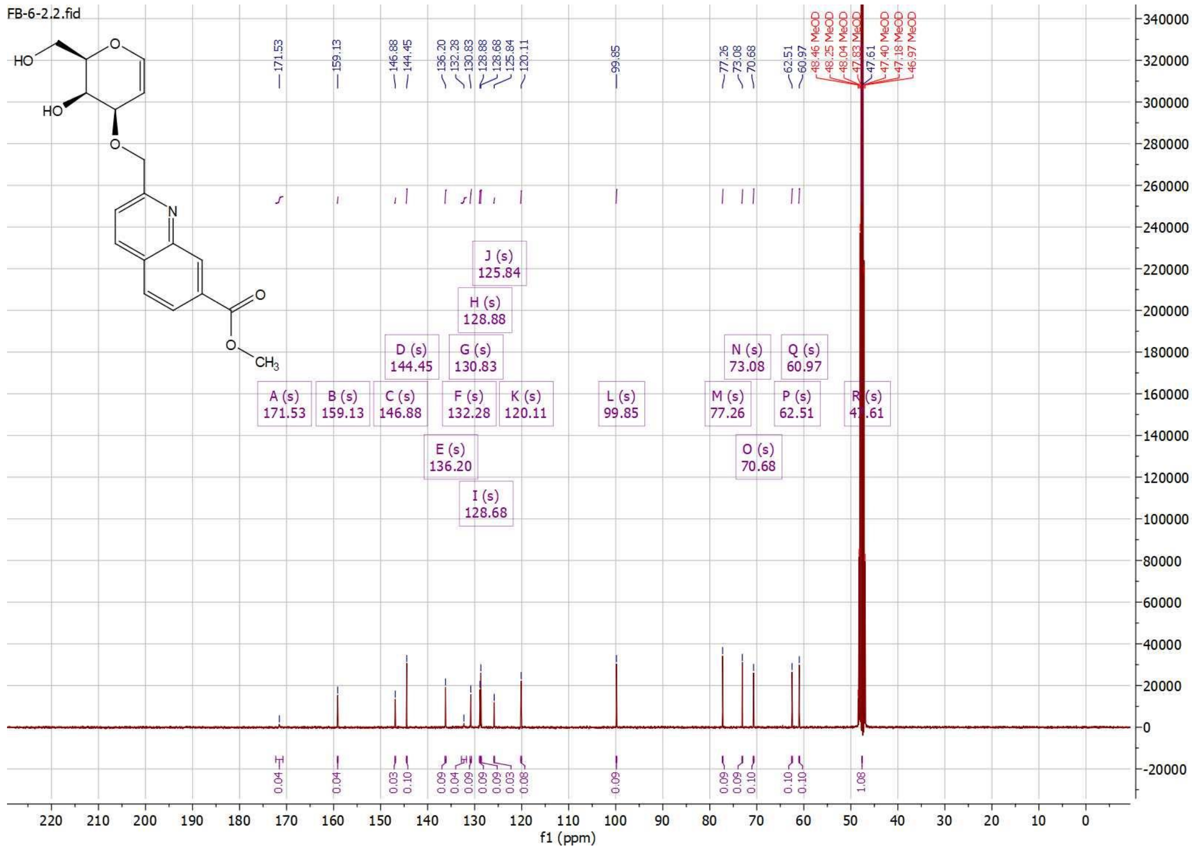

Supplement: Supplementary file 1 — ml1c00371_si_001.pdf [file ml1c00371_si_001.pdf]
